# Design of Cytotoxic T Cell Epitopes by Machine Learning of Human Degrons

**DOI:** 10.1021/acscentsci.3c01544

**Published:** 2024-03-06

**Authors:** Nicholas L. Truex, Somesh Mohapatra, Mariane Melo, Jacob Rodriguez, Na Li, Wuhbet Abraham, Deborah Sementa, Faycal Touti, Derin B. Keskin, Catherine J. Wu, Darrell J. Irvine, Rafael Gómez-Bombarelli, Bradley L. Pentelute

**Affiliations:** † Department of Chemistry, Massachusetts Institute of Technology, Cambridge, Massachusetts 02139, United States; ‡ Department of Chemistry and Biochemistry, University of South Carolina, Columbia, South Carolina 29208, United States; § Department of Materials Science and Engineering, Massachusetts Institute of Technology, Cambridge, Massachusetts 02139, United States; ∥ Machine Intelligence and Manufacturing Operations Group, Massachusetts Institute of Technology, Cambridge, Massachusetts 02139, United States; ⊥ The Koch Institute for Integrative Cancer Research, Massachusetts Institute of Technology, Cambridge, Massachusetts 02142, United States; # Ragon Institute of Massachusetts General Hospital, Massachusetts Institute of Technology, and Harvard University, Cambridge, Massachusetts 02139, United States; & Department of Medical Oncology, Dana-Farber Cancer Institute, Boston, Massachusetts 02215, United States; ¶ Harvard Medical School, Boston, Massachusetts 02115, United States; □ Broad Institute of MIT and Harvard, Cambridge, Massachusetts 02142, United States; ■ Translational Immunogenomics Laboratory (TIGL), Dana-Farber Cancer Institute, Boston, Massachusetts 02215, United States; ○ Department of Computer Science, Metropolitan College, Boston University, Boston, Massachusetts 02215, United States; △ Section for Bioinformatics, Department of Health Technology, Technical University of Denmark, Lyngby DK-2800, Denmark; ▲ Department of Medicine, Brigham and Women’s Hospital, Boston, Massachusetts 02115, United States; ▽ Department of Biological Engineering, Massachusetts Institute of Technology, Cambridge, Massachusetts 02139, United States; ▼ Howard Hughes Medical Institute, Chevy Chase, Maryland 20815, United States; ⬡ Center for Environmental Health Sciences, Massachusetts Institute of Technology, Cambridge, Massachusetts 02139, United States

## Abstract

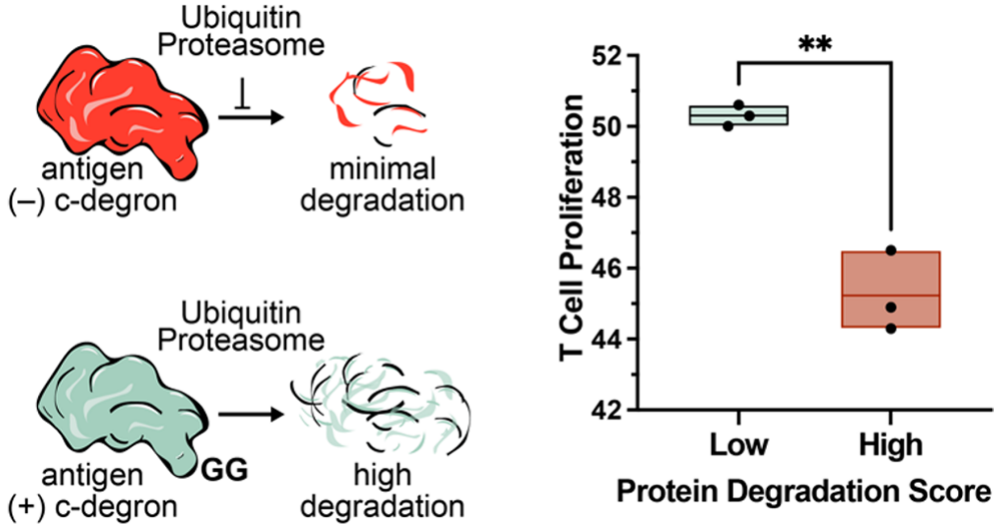

Antigen processing is critical for therapeutic vaccines to generate epitopes for priming cytotoxic T cell responses against cancer and pathogens, but insufficient processing often limits the quantity of epitopes released. We address this challenge using machine learning to ascribe a proteasomal degradation score to epitope sequences. Epitopes with varying scores were translocated into cells using nontoxic anthrax proteins. Epitopes with a low score show pronounced immunogenicity due to antigen processing, but epitopes with a high score show limited immunogenicity. This work sheds light on the sequence–activity relationships between proteasomal degradation and epitope immunogenicity. We anticipate that future efforts to incorporate proteasomal degradation signals into vaccine designs will lead to enhanced cytotoxic T cell priming by these vaccines in clinical settings.

## Abstract 2

Design of cytotoxic T cell epitopes for enhancing antigen processing and presentation, enabled by machine learning of human degrons and cytosolic delivery with two anthrax proteins.

## Introduction

Vaccines are transforming human health through enabling a patient’s own immune system to defend against cancer and pathogenic disease.^1,2^ The components of a vaccine aim to mimic the biological processes associated with acquiring natural immunity by generating immune cell populations that recognize tumor- and pathogen-specific epitopes.^3^ Vaccine formulations that prime new and existing T cell populations offer the promise of not only eliminating affected cells but also providing long-term protection through immune-memory responses.^4^ Despite this promise, the design and development of vaccine epitopes that provide robust priming of cytotoxic T cells has thus far proved difficult. One key challenge is thought to be due to insufficient epitope processing and presentation, which leads to reduced vaccine efficacy.^5^

**Figure 1:**
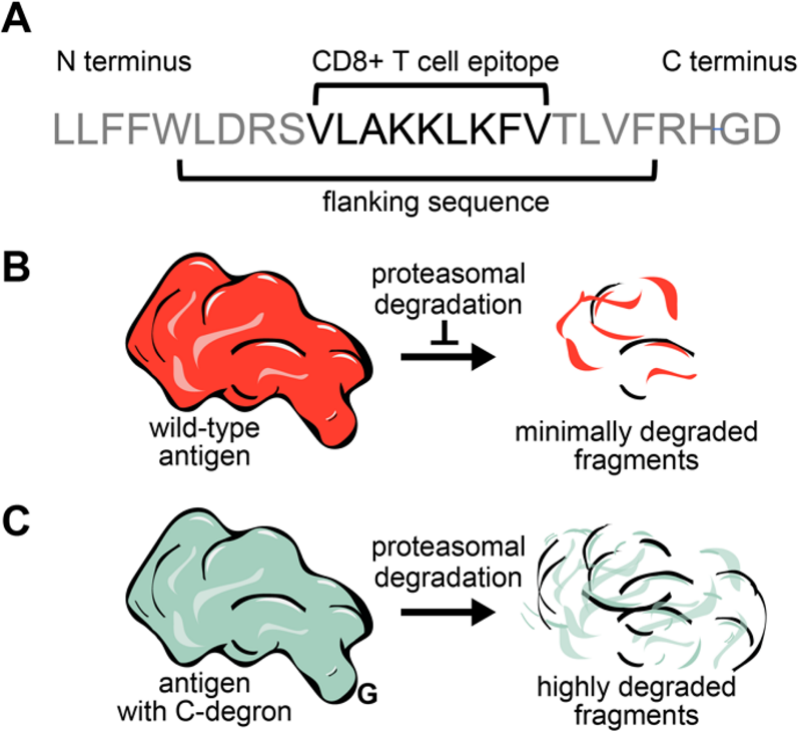
Vaccine design with human degrons. (A) Representative immunizing peptide, which comprises a cytotoxic (CD8+) T cell epitope and a flanking sequence. (B) Wild-type antigens that do not contain a degron sequence undergo minimal processing. (C) Antigens that contain a C-terminal degron (C-degron) sequence undergo pronounced processing, resulting in enhanced epitope presentation by HLA molecules and the priming of cytotoxic T cells.

Developing an antigen-specific vaccine typically includes sequence characterization of a tumor, bacterial, or viral protein, followed by the design of one or more immunogenic epitopes for the vaccine formulation. The epitope sequence typically comprises 8–9 amino acids for cross-presentation and additional flanking amino acids to limit uncontrolled proteolysis. An ideal immunization epitope undergoes cross-presentation by MHC class I molecules to prime cytotoxic CD8+ T cells, in addition to MHC class II presentation of longer sequences to prime immune-memory CD4+ T cells (Figure 1A).^6−10^ The design of these epitopes is aided by immunopeptidomics combined with computational tools, which enable optimization of MHC-binding affinity^11−13^ and T cell receptor (TCR) specificity.^14^ Although these tools enable design of epitopes that can be presented, particularly for personalized cancer vaccines,^15^ they provide little insight into designing the flanking residues. These flanking residues are particularly important, because a single mutation can alter the magnitude of antigen processing and presentation for a given epitope.^16^

Antigen cross-presentation is dependent on protein processing in the cytosol of antigen-presenting cells, followed by the transport to the endoplasmic reticulum and loading onto MHC class I molecules.^17^ Early efforts to facilitate processing include incorporation of protease cleavage sites by cathepsin and furin proteases,^18^ but these enzymes also mediate endosomal processing which favors class II presentation.^19^ Recent efforts to facilitate cytosolic processing include the incorporation of ubiquitin fusion proteins^20,21^ and proteolysis-targeting chimeras (PROTACs).^22^ Although these efforts enhance immunogenicity, the rules to predict processing and presentation for a given epitope remain to be uncovered.

Intracellular degradation of peptides and proteins is prevalent in cellular metabolism and may provide rules for designing vaccine epitopes and other therapeutic polypeptides.^23^ The degradation is based on short peptide sequences, called degrons, that signal the proteasome for protein degradation.^24^ In 1986, Varshavsky and co-workers discovered that a single amino acid at the N-terminus can dictate the propensity of proteasomal degradation, which is now known as the “N-end rule.”^25^ In 2015, we similarly observed that a single d-amino acid can abrogate proteasomal degradation, perhaps due to the absence of ubiquitin ligases that can recognize d-amino acids.^26^ Moreover, Elledge and co-workers demonstrated that longer stretches of amino acids, up to 23 residues in length, adopt sequence patterns at either the N or C terminus that greatly influence the magnitude of proteasome degradation.^27−32^ Machine learning studies recently showed that degron sequences can prolong the lifetimes of oncoproteins in cancer,^33^ which suggests that degrons may also influence antigen processing and presentation (Figure 1B, C).

In this work, we set out to uncover vaccine design rules that infer epitope immunogenicity from proteasomal degradation activity. We used machine learning to create our own degron prediction model to enable interpretation of complex antigen sequence patterns and their proteasomal stabilities. The training data comprises the proteasomal stabilities of 22 564 sequences from the C-termini of human proteins and reflects the propensity of protein degradation across the human proteome. The resulting model ascribes a relative degradation score between 0 and 100, which is determined from the C-terminal residues of a peptide or protein sequence. The model does not include stability data from N-terminal sequences because multiple nonproteasome mechanisms are known to hydrolyze the N-terminus, including N-terminal trimming from an endoplasmic reticulum aminopeptidase (ERAP) 1 or 2.^34^

To validate our C-degron model, we used a protein delivery system that efficiently transports epitope peptides into antigen-presenting cells to ensure cytosolic delivery and provide access to the proteasomal degradation machinery. We used two nontoxic anthrax proteins for epitope translocation: protective antigen (PA) and the N-terminus of lethal factor (LF_N_). Incorporating C-degron peptides at the C-terminus of LF_N_ enabled validation of predicted proteasomal degradation activity by Western blot analysis and a series of T cell proliferation assays. These studies show that combining C-degron sequences with epitope peptides favors proteasomal degradation and, in turn, maximizes epitope immunogenicity.

## Results

### Machine Learning for Prediction of Human Degrons

**Figure 2:**
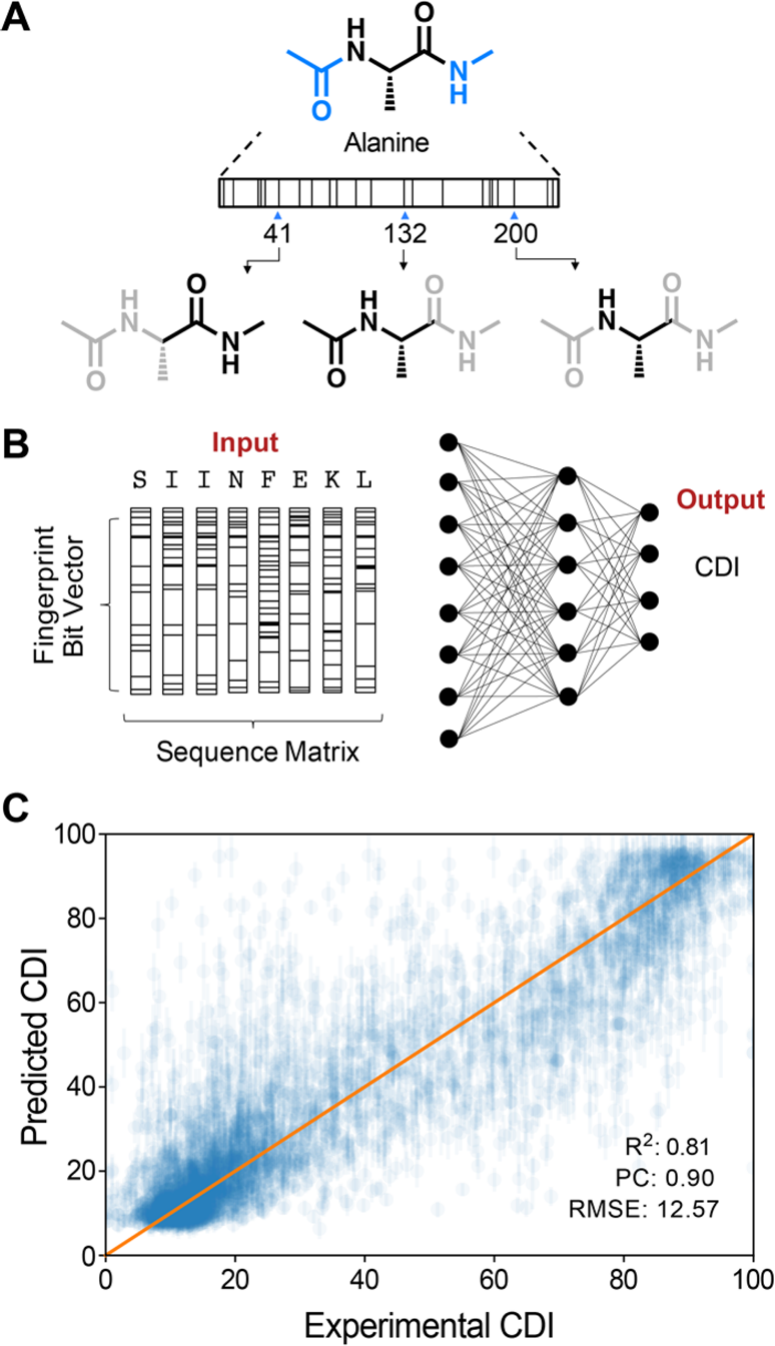
Machine learning of human degron sequences. (A) Fingerprint representation of a single amino acid. (B) Sequence matrix representation for the SIINFEKL epitope of ovalbumin_257–264_, illustrating the matrix inputs and outputs for predicting C-terminal degron index (CDI). (C) Observed versus predicted parity plots for linear regression analysis of CDI. Training data were split into three subsets for model training (60%), cross-validation (20%), and testing (20%). Inset text and graphics indicate key performance metrics: *R*^2^, Pearson’s correlation (PC), root-mean-squared error (RMSE), and solid orange line, *y* = *x* line.

Previously, we demonstrated that deep neural network models can be trained to relate peptide sequences to biological activity and to aid design loops for developing novel bioactive peptides with complex design principles.^35^ Our strategy relies on training one-dimensional convolutional networks on peptide sequences, by representing the monomer identity as a fingerprint that reflects the chemical structure (Figure 2A).^36,37^ The models are interrogated using random sequences to permit experimental validation and further optimization.^38,39^ Here, we use this strategy to correlate amino acid sequences with their degradation propensity.

The training data were obtained from stability index studies previously described by Elledge and co-workers, comprising a plasmid library of DNA-encoded peptides from the human proteome.^31^ The peptides include the 23 residues from the C-termini of human protein sequences which are fused to the green fluorescent protein (GFP). In prior work, transfection of these DNA libraries into mammalian cells demonstrated protein degradation from the GFP expression levels. Experimental data revealed a distribution of fluorescent cells among numeric “bins” (e.g., bin1, bin2, bin3, and bin4). The fraction of cell populations in the lower bins (e.g., bin1) indicate reduced fluorescence intensity associated with GFP degradation; the fraction of cell populations in the higher bins (e.g., bin4) indicate high fluorescence intensity associated with intact GFP.

We developed a linear equation that approximates the proteasomal stability data to a single score, which we call the C-terminal Degron Index (CDI). This score is calculated from a linear combination of two parameters: bin population data (e.g., bin1, bin2, bin3, and bin4) and exponential coefficients (e.g., 0, 1, 10, and 100), which reflect the exponential scale of the original data (i.e., flow cytometry). The resulting score relates degradation propensity to a numerical CDI that ranges from 0 to 100. Interpreting the CDI for a given sequence is straightforward: a CDI value in the lower quartile (i.e., 0–25) reflects pronounced degradation; a CDI value in the upper quartile (i.e., 75–100) reflects limited degradation. The CDI also enables comparisons of closely related values (e.g., 20 vs 25) and their corresponding degradation propensity.

To prepare the input data for machine learning, we calculated CDI values across the human proteome using the sequence data (22 564 sequences). The machine learning model was established using 100% of the input sequences and CDI values; however, the model was trained, validated, and tested using the following three randomized subsets of the data: 60%, 20%, and 20%, respectively (Figure 2B). On a 20% subset, we calculated several measures of statistical significance by fitting the data to the CDI model. The resulting analysis showed the following: root-mean-squared error (RMSE), CDI_RMSE_ = 12.97 ± 0.11; linear regression *R*-squared value, *R*^2^ = 0.796 ± 0.003; and Pearson correlation coefficient, ρ = 0.896 ± 0.002 (Figure 2C). These values show that the CDI model can reasonably ascribe degradation propensity to a given sequence located at the C-terminus of a peptide or protein.

**Figure 3:**
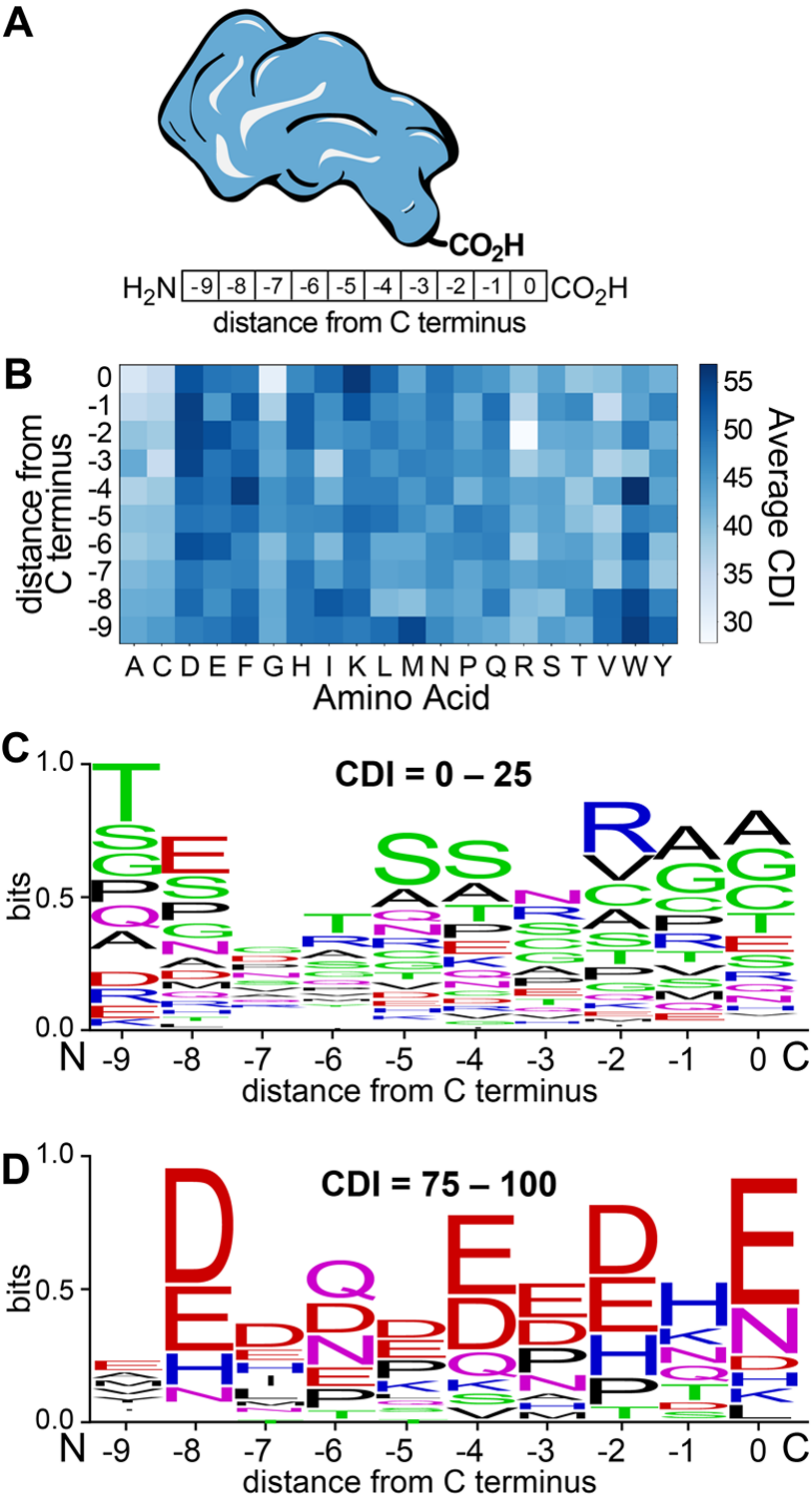
Interrogating the C-degron prediction model. (A–D) Analysis of randomized sequences (30 000) based on the last 10 C-terminal amino acids on a protein. (A) Illustration of the C-terminal residues (10 amino acids) that influence proteasomal stability. (B) Heat map plot showing the C-terminal amino acids (10 amino acids) and average CDI values (ranging from 30 to 55). (C, D) Sequence logo plots of recurrent amino acids at corresponding positions, which were plotted across CDI values for lower (CDI = 0–25) and upper (CDI = 75–100) quartiles.

We interrogated the model by generating a randomized sequence library and analyzing sequence trends based on CDI. The library was obtained from 30 000 randomly generated sequences that vary at the C-terminus (i.e., 10 residues) but maintain a constant N-terminus (Figure 3A). We generated a heat map to evaluate sequence trends that emerge for the 10 C-terminal residues (i.e., positions 0 to −9) (Figure 3B). We also generated sequence logo plots for two key populations, in which one plot reflects the lower quartile (CDI: 0–25, Figure 3C) and the other reflects the higher quartile (CDI: 75–100, Figure 3D).

The heat map and sequence logo plots reveal identities and positions of amino acids that favor degradation. Consistent with prior experiments, the model predicts that a Gly residue located at position 0 or −1 contributes substantially to degradation propensity (i.e., CDI ≤ 30); the model also predicts that a Gly residue located further from the C terminus (i.e., position −6 or −7) also favors proteasomal degradation but to a lesser extent. The plots show that other amino acid residues are also predicted to favor degradation (i.e., CDI ≤ 40): an Ala residue at position 0, −1, −2, −4, −5, −6, or −7; a Cys residue at position 0, −1, −2, −3, −4, −5, or −6; an Ile residue at −3 or −6; an Arg residue at position 0, −1, −2, −3, −6, −8, or −9; a Ser residue at position −3 or −8; a Thr residue at position 0, −4, or −5; and a Val residue at position 0, −1, −2, −3, −5, −6, or −7. These amino acid residues are thought to reflect substrates of ubiquitin ligase enzymes, which mediate ubiquitin ligation and subsequent proteasomal degradation.

The model also predicts amino acid residues that mostly do not favor degradation (i.e., CDI > 40); these residues include Asp, Glu, Asn, Gln, Phe, Pro, His, Lys, Leu, Met, Trp, and Tyr. The heat map and sequence logo plots shed light on the identities and positions of residues that do not favor degradation (Figure 3B, D). Nonetheless, CDI is determined from an overall sequence rather than the individual residues, and therefore, a sequence may still give a low CDI while containing one or more residues that do not favor degradation.

### Predicted Degrons Regulate Proteasomal Degradation

Bacterial toxin proteins have previously been shown to enable protein degradation studies, because these studies are otherwise notoriously challenging without ensuring cytosolic delivery.^40^ Here, two anthrax proteins were used for cytosolic epitope delivery: PA and LF_N_. Previously, PA/LF_N_ have been shown to enable cytosolic delivery of cytotoxic T cell epitopes.^41,42^ The proteins mediate translocation through PA binding to a transmembrane protein receptor, either tumor endothelial cell marker (TEM8) or capillary morphogenesis protein 2 (CMG2), and mediate the delivery of lethal and edema factors into the cytosol of mammalian cells.^43,44^

**Figure 4:**
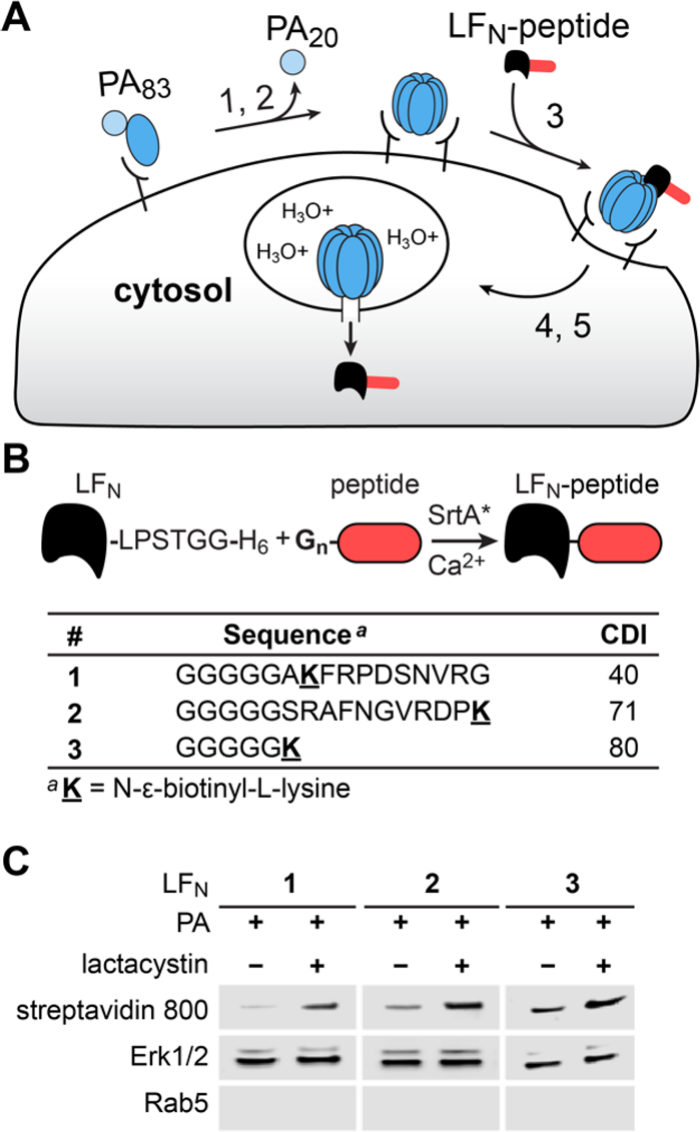
Proteasomal stability reflects the magnitude of protein degradation. (A) Illustration of translocation by protective antigen (PA) and the N-terminus of lethal factor (LF_N_). (B) Peptide incorporation onto the C-terminus of LF_N_ by sortase-mediated ligation and sequences of biotinylated peptides **1**–**3**. (C) Western blot analysis after PA-mediated translocation of LF_N_**1**–**3** in CHO-K1 cells, with and without pretreatment of cells with 20 μM lactacystin.

In the current study, peptides were conjugated to the C-terminus of LF_N_ using sortase-mediated ligation (Figure 4A).^45^ These LF_N_–peptide conjugates were coadministered with the pore-forming protein, PA, enabling cytosolic delivery through a multistep mechanism.^46^ The PA-mediated translocation mechanism is well established, which includes the following: (1) binding to receptors on mammalian cells; (2) undergoing cleavage to give 20-kDa and 63-kDa fragments, called PA_20_ and PA_63_, followed by PA_63_ assembling to form annular heptamers (PA_63_)_7_; (3) further assembling with LF_N_ molecules; (4) entering the cell endosome; and (5) rearranging for insertion into the endosomal membrane for LF_N_ translocation into the cell.

We measured the presence of translocated LF_N_ protein to evaluate whether proteasomal degradation is favored or disfavored based on CDI. For these studies, we combined LF_N_ with synthetic peptides **1**–**3** that show varying CDI values: 40, 71, and 80 (Figure 4B). Proteasomal degradation was evaluated with CHO-K1 cells, which is an established cell line that expresses anthrax receptors and enables PA-mediated binding and cytosolic protein translocation. The peptides were evaluated with and without pretreating CHO cells with the lactacystin proteasome inhibitor, followed by incubating the cells with PA (20 nM) and LF_N_**1**–**3** (100 nM). Cytosolic extraction with a digitonin buffer, followed by Western blot analysis, established the cytosolic fraction based on the presence of horizontal bands associated with ERK1/2, which are well-known MAP kinase proteins that are located in the cytosol (Figure 4C).^47^ The cytosolic fraction was further established by the absence of a Rab5 band, which is a key protein that localizes in early endosomes.^48^ Proteasomal degradation of LF_N_**1**–**3** was established based on the intensity of horizontal bands associated with biotinylated protein, which become darker after lactacystin treatment. These studies establish that PA/LF_N_ successfully delivers peptides **1**–**3** into CHO-K1 cells and that peptide degradation occurs in a proteasome-dependent fashion.

### Automated Flow Synthesis of Vaccine Epitopes

We used automated flow peptide synthesis (AFPS) to accelerate studies that relate proteasomal stability to epitope immunogenicity.^49^ We synthesized antigen peptides derived from ovalbumin (OVA) that contain the OVA_257–264_ (SIINFEKL) epitope to impart immunogenicity.^50,51^ We designed 20 peptide variants, which were prepared and conjugated to LF_N_ (Table 1). The peptides each comprise three N-terminal Gly residues for sortase-mediated ligation, native epitope residues from OVA_257–264_ (SIINFEKL) epitope for immunogenicity, and varying flanking residues for tuning degradation activity. A subset of eight peptides, called OVA **1**–**8**, contain native resides but only differ in length (13–28 amino acids); a subset of four peptides, called OVA **9**–**12**, contain mutated flanking residues that impart a high score (i.e., CDI > 50). Another subset of eight peptides, called OVA **1G**–**8G**, are homologues of OVA **1**–**8** but contain two additional Gly residues that impart a low score (i.e., CDI < 25). Altogether, the OVA **1**–**12** and **1G**–**8G** peptides permit comparisons of degradation propensity and immunogenicity, without conflicting influences arising from physical properties such as aromaticity, isoelectric point, net charge, and secondary structure (Table S1). The peptides were prepared by AFPS on HMPB-ChemMatrix resin, cleaved from the resin under acidic conditions, and purified by reverse phase (RP)-HPLC. Mass spectrometry (ESI) analysis showed the desired mass for the resulting LF_N_-OVA fusion proteins after sortase-mediated ligation (Figures S1–S20).

**Table 1:** Summary of Ovalbumin (OVA) **1**–**12** and **1G**–**8G** Peptides

peptide	description	sequence	CDI
OVA **1**	G_3_ + OVA_252–264_	GGGLEQLESIINFEKL	40
OVA **2**	G_3_ + OVA_252–265_	GGGLEQLESIINFEKLT	37
OVA **3**	G_3_ + OVA_252–266_	GGGLEQLESIINFEKLTE	44
OVA **4**	G_3_ + OVA_252–267_	GGGLEQLESIINFEKLTEW	27
OVA **5**	G_3_ + OVA_252–268_	GGGLEQLESIINFEKLTEWT	28
OVA **6**	G_3_ + OVA_252–269_	GGGLEQLESIINFEKLTEWTS	20
OVA **7**	G_3_ + OVA_252–270_	GGGLEQLESIINFEKLTEWTSS	27
OVA **8**	G_3_ + OVA_252–279_	GGGLEQLESIINFEKLTEWTSSNVMEERKIK	35
OVA **9**	G_3_ + OVA_257–264_ + generated	GGGTSIINFEKLTECTSSNVMEERK	53
OVA **10**	G_3_ + OVA_257–264_ + generated	GGGEQLEESIINFEKLTN	58
OVA **11**	G_3_ + OVA_257–264_ + generated	GGGEHEESIINFEKLTETKDPETE	88
OVA **12**	G_3_ + OVA_257–264_ + generated	GGGEPSIINFEKLEDQKESNNEDNEDK	99
OVA **1G**	G_3_ + OVA_252–264_ + GG	GGGLEQLESIINFEKLGG	19
OVA **2G**	G_3_ + OVA_252–265_ + GG	GGGLEQLESIINFEKLTGG	14
OVA **3G**	G_3_ + OVA_252–266_ + GG	GGGLEQLESIINFEKLTEGG	25
OVA **4G**	G_3_ + OVA_252–267_ + GG	GGGLEQLESIINFEKLTEWGG	10
OVA **5G**	G_3_ + OVA_252–268_ + GG	GGGLEQLESIINFEKLTEWTGG	14
OVA **6G**	G_3_ + OVA_252–269_ + GG	GGGLEQLESIINFEKLTEWTSGG	14
OVA **7G**	G_3_ + OVA_252–270_ + GG	GGGLEQLESIINFEKLTEWTSSGG	17
OVA **8G**	G_3_ + OVA_252–279_ + GG	GGGLEQLESIINFEKLTEWTSSNVMEERKIKGG	19

### Processing and Presentation of Translocated Antigens Are Proteasome Dependent

**Figure 5:**
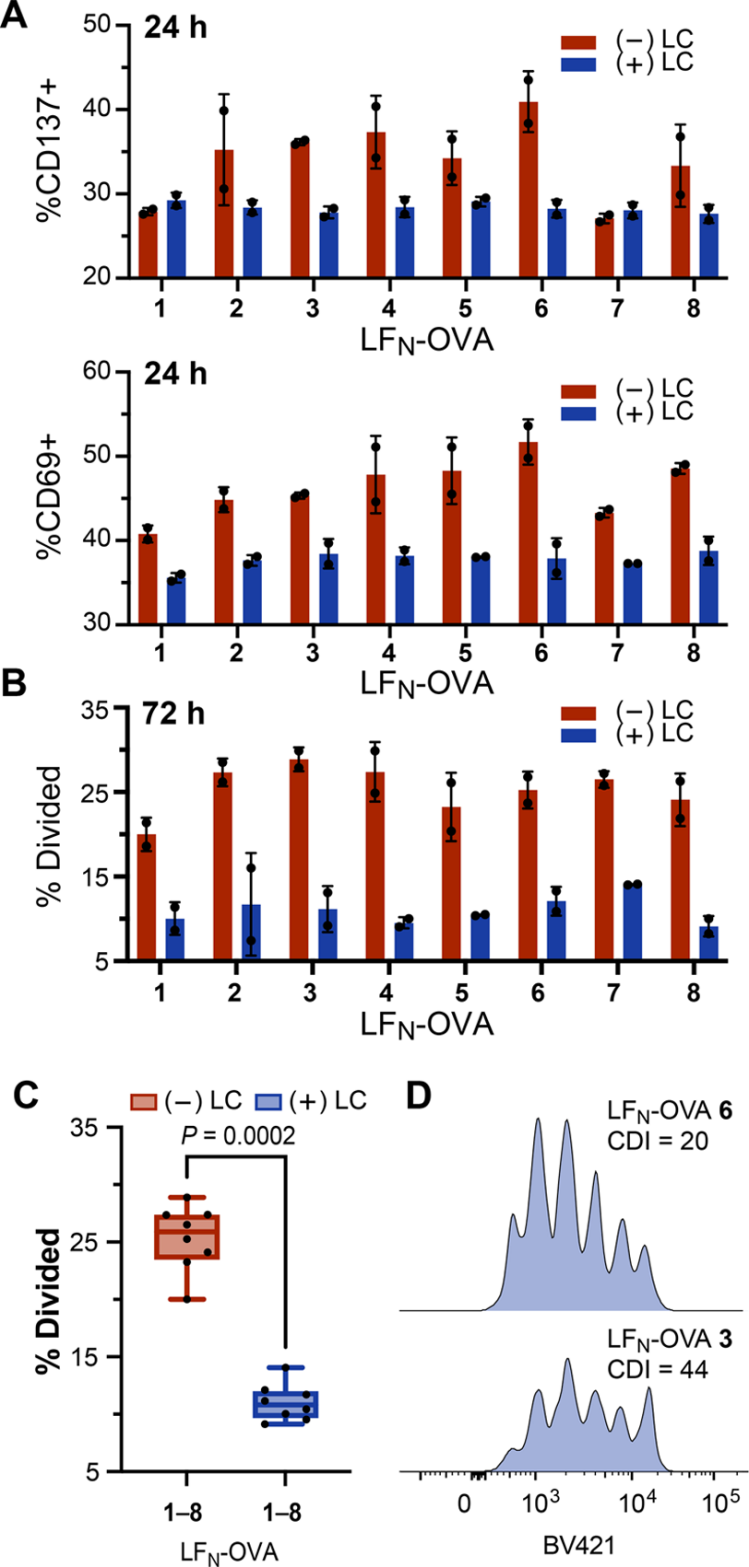
Proteasomal degradation of translocated protein regulates T cell proliferation. (A–D) Flow cytometry analysis of Cell-Trace Violet (BV421)-labeled T cells (Thy1.1^+^ OT-1) after incubation with primary C57BL/6 dendritic cells (DCs). DCs were prepared by lactacystin (LC) treatment, with (+) or without (−), followed by incubation with PA (20 nM) and LF_N_-OVA **1**–**8** (1 μM). (A) Plot of CD137+ and CD69+ cells after 24 h. (B) Plot of cell proliferation (% divided) after 72 h. (C) Grouped pairwise comparison of responses from Figure 5B. Data represent the mean, minimum, and maximum. Statistical analysis is a Mann–Whitney test, which indicates the comparison is significantly different. (D) Representative histogram plots from PA-translocation of LF_N_-OVA **3** and **6**. Data are representative of three independent experiments.

We used the PA/LF_N_-OVA constructs to evaluate the influence of a degron sequence on immunogenicity. Primary dendritic cells (DCs) from mouse splenocytes (C57BL/6) were obtained, treated with lactacystin (20 μM) for 1 h, and coincubated with PA/LF_N_-OVA constructs. Coincubating the DCs with CellTrace Violet-labeled T cells (OT-1) followed by flow cytometry analysis revealed T-cell activation (24 h) based on upregulation of CD69 and CD137 markers (Figure 5A, Figure S21) and also T-cell proliferation (72 h) based on dilution of the dye (Figure 5B, Figure S22).

Immunogenicity studies with the LF_N_-OVA epitopes established that T cell proliferation is dependent on proteasomal degradation (i.e., CDI). The proteasome dependence is reflected by diminished T cell activation in the presence of a proteasome inhibitor. The LF_N_-OVA epitopes were evaluated by preincubating murine DCs with and without 100 μM lactacystin, followed by evaluating proliferation of OVA-specific T cells. Grouping the activities from the LF_N_-OVA **1**–**8** constructs, with and without treatment of lactacystin, showed a significant decrease in immunogenicity due to the inhibited proteasome (Figure 5C). Moreover, several LF_N_-OVA constructs showed pronounced differences in T cell proliferation activity, even though the protein concentrations and SIINFEKL epitopes were identical, which suggests differences in the magnitude of proteasomal processing activity (Figure 5D).

### Proteasomal Stability Reflects T Cell Epitope Immunogenicity

Titrating the LF_N_-OVA concentration revealed a dependence of concentration on proteasome-mediated immunogenicity. This dependence is reflected by varying the concentrations of LF_N_-OVA (10, 1, and 0.1 μM) while maintaining a constant concentration of PA (20 nM). To facilitate comparison, we excluded LF_N_-OVA **1** due to the absence of epitope flanking residues, which can circumvent processing, and excluded LF_N_-OVA **8** due to the presence of extended flanking residues, which can undergo proteolytic and proteasomal processing.

**Figure 6:**
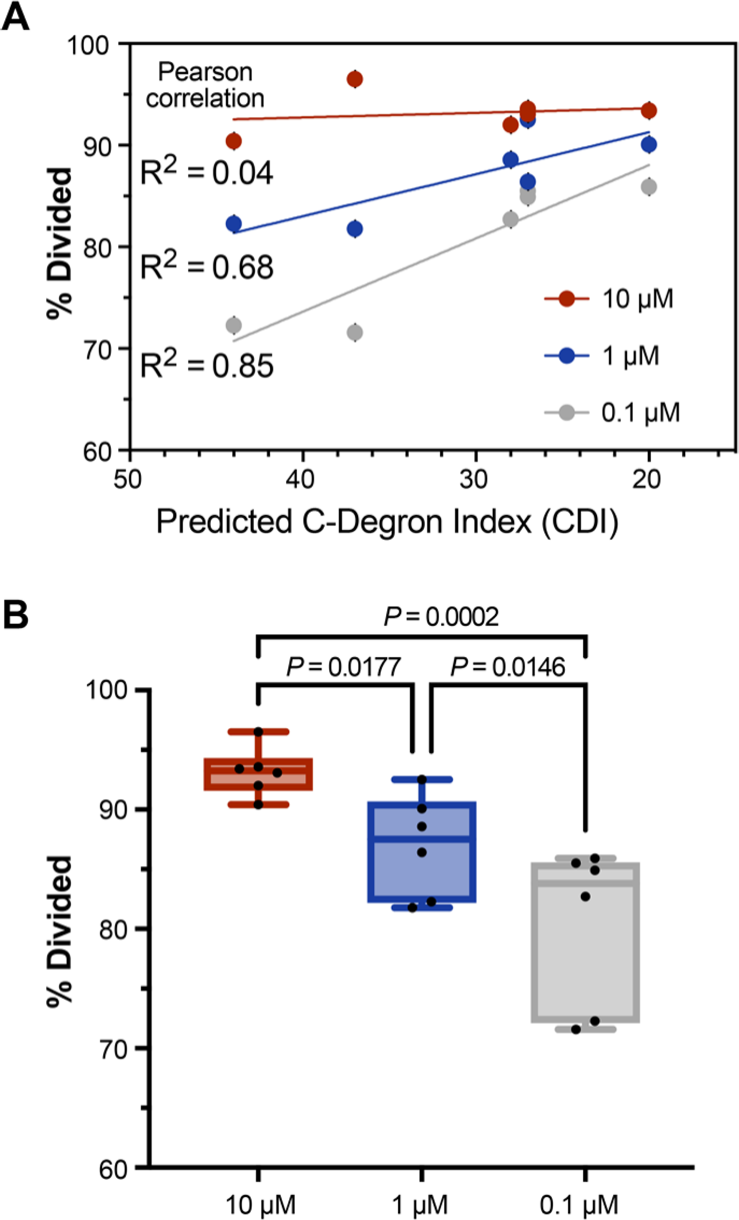
Proteasomal stability influences the magnitude of T cell proliferation. (A) Plot of T cell proliferation (% divided) against the C-degron index (CDI) after incubation with murine DCs. DCs were prepared by treatment with PA (20 nM) and LF_N_-OVA **2**–**7** (10, 1, and 0.1 μM). Statistical analyses are Pearson correlation tests. (B) Grouped comparisons of the responses from (A). Statistical analyses are uncorrected Fisher’s least-squared difference tests, which indicate the comparisons are significantly different. Data are representative of three independent experiments.

Treatment of primary DCs with PA/LF_N_-OVA **2**–**7** showed that the magnitude of T cell proliferation is dependent on concentration and CDI. At the lowest concentration (0.1 μM), LF_N_-OVA epitopes show a strong correlation (*R*^2^ = 0.85) between CDI and T cell proliferation (% divided). At higher concentrations (1 and 10 μM), the correlation steadily decreases (*R*^2^ = 0.68 and 0.04, respectively) due to saturation of the antigen-presenting cells (Figure 6A, B). These results suggest that degron activity is important not only for tuning epitope immunogenicity but also for maximizing epitope efficacy at lower concentrations.

**Figure 7:**
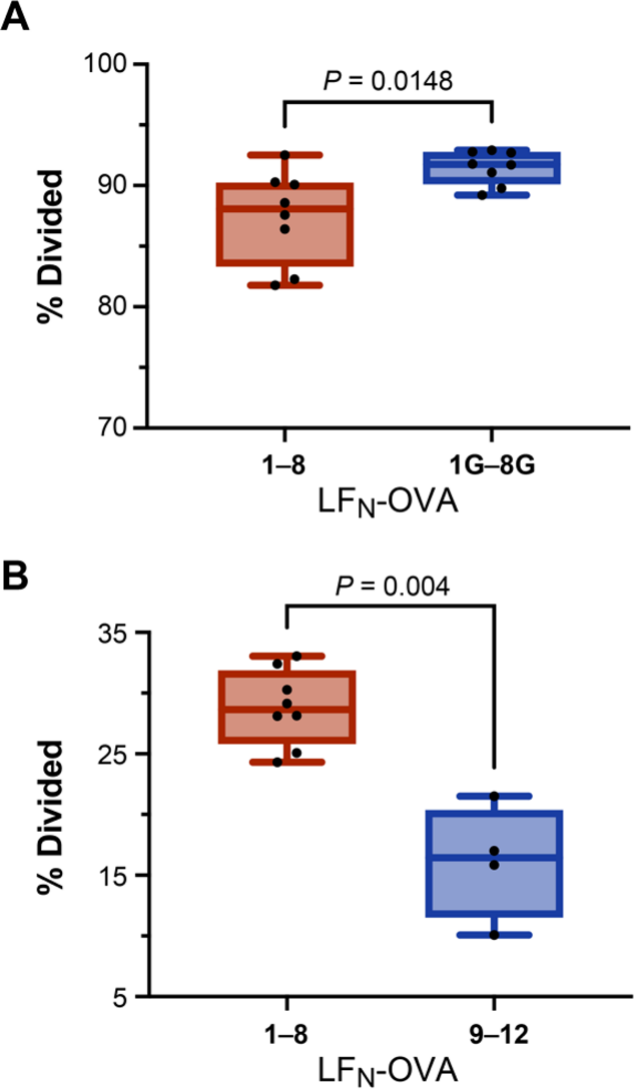
Varying proteasomal stability alters the magnitude of T cell proliferation. Plots of OT-1 T cell proliferation (% divided) after incubation with murine DCs. DCs were prepared by treatment with PA (20 nM) and LF_N_-OVA **1**–**8**, LF_N_-OVA **1G**–**8G**, or LF_N_-OVA **9**–**12** (1 μM). Statistical analyses are Mann–Whitney tests, which indicate the comparisons are significantly different. Data are representative of at least three independent experiments.

Mutating the flanking residues of the OVA epitope further reveals the influence of proteasomal degradation. LF_N_-OVA **1**–**8** show modest T cell proliferation activity that varies between each construct. LF_N_-OVA **1G**–**8G** show pronounced T cell proliferation that is nearly identical across all eight sequences (Figure 7A), and LF_N_-OVA **9**–**12** show limited T cell proliferation (Figure 7B). The mutated OVA sequences further demonstrate the influence of CDI on immunogenicity. Although the T cell epitope is identical across all sequences, the activity varies for both the native and mutated sequences. These variations appear to reflect the predicted CDI, in which the magnitude of T cell proliferation greatly depends on whether degradation is predicted to increase or decrease.

### Tuning Proteasomal Stability Enhances Immunogenicity

**Figure 8:**
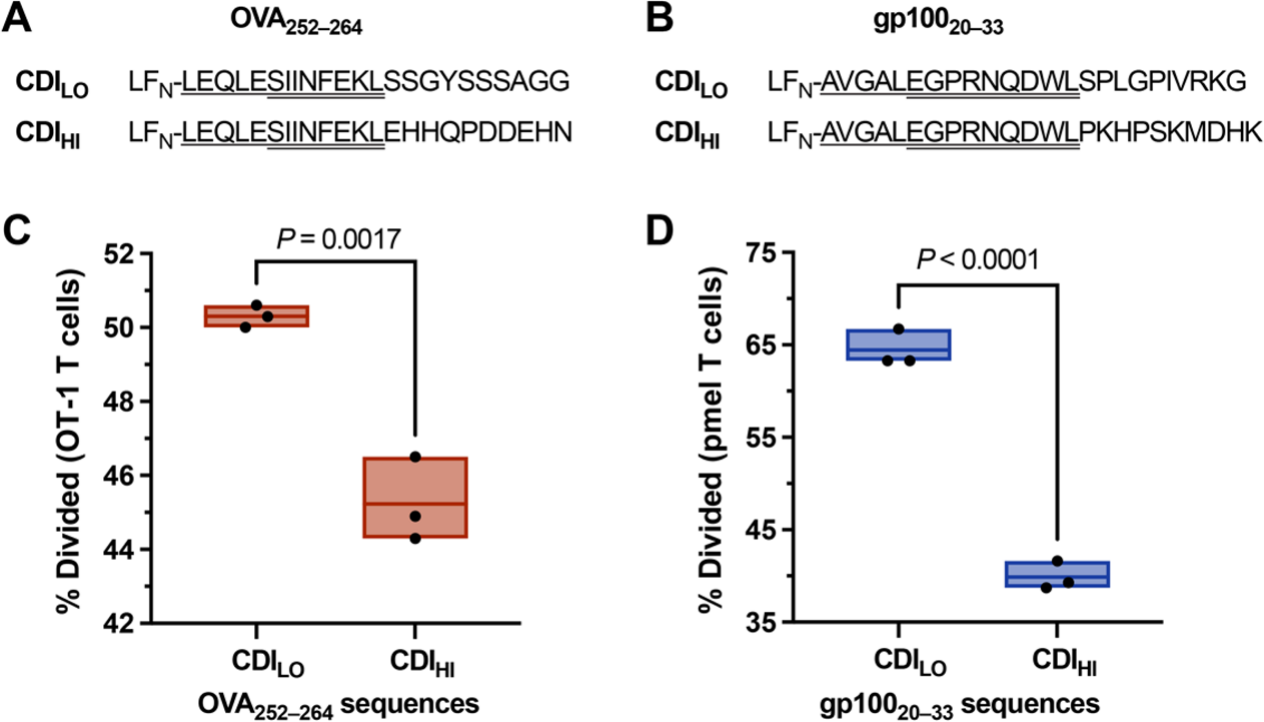
Promoting proteasomal degradation enhances epitope immunogenicity. (A, B) Epitope sequences (8–9 amino acids) with varying proteasomal stabilities (CDI) adopted from ovalbumin (OVA_252–264_) and glycoprotein 100 (gp100_20–33_). Each sequence comprised the native residues from the N-terminal (bolded) and epitope (underlined) regions, followed by randomized C-terminal residues (10 amino acids). The ten randomized residues were generated using a ML goal-search algorithm, which afforded diverse sequences (30 000-membered peptide library) with varying proteasomal stabilities, including CDI = <10 (CDI_LO_) and >60 (CDI_HI_). Peptides from each group were selected, synthesized with three N-terminal Gly residues (i.e., GGG-peptide), and conjugated to LF_N_ with SrtA*. (C, D) Plots of T cell proliferation (% divided) after incubation with murine DCs. DCs were prepared by treatment with PA (20 nM) and the indicated LF_N_-CDI_LO_ or LF_N_-CDI_HI_ (1 μM). Statistical analyses are unpaired *t* tests, which indicate that the comparisons are significantly different. Data are representative of at least three independent experiments.

We further evaluated degron sequences through binary comparisons of high and low degradation activities. These comparisons shed light on epitope immunogenicity for other disease models (Figure 8). For each epitope, we generated randomized C-terminal sequences using a machine learning-based goal-search algorithm, in which the randomized sequences consist of 30 000-membered libraries. Each peptide comprises the native epitope sequences (8–9 amino acids) and N-terminal (5 amino acids) residues but also contain randomized C-terminal residues (10 amino acids). Peptides were selected from the library that exhibit low (CDI_LO_) and high (CDI_HI_) degradation scores (Figures S23–S24). Although physical properties varied between these peptides (Table S2), we anticipated the CDI values would dictate immunogenicity. LF_N_ conjugates were prepared by synthesizing the CDI_LO_ and CDI_HI_ peptides using automated flow peptide synthesis, followed by purification and conjugation to LF_N_ (Figures S25–S28).

Relative immunogenicity of the CDI_LO_ and CDI_HI_ peptides was evaluated with primary DCs (C57BL/6). The DCs were treated with PA and the LF_N_-CDI_LO_ and LF_N_-CDI_HI_ conjugates, followed by coincubation (72 h) with CellTrace Violet-labeled T cells obtained from transgenic mouse models: OT-1 and human premelanosome protein (pmel). Flow cytometry analysis of the T cells revealed CDI-dependent proliferation from the translocated epitopes into DCs: CDI_LO_ peptides show pronounced proliferation, and CDI_HI_ epitopes show limited proliferation. This comparison shows that reducing the CDI can increase epitope-specific T cell proliferation through favoring proteasomal degradation.

## Discussion

Although proteasomal degradation is an established step in the antigen-processing pathway, several limitations have precluded the development of epitope design rules thus far. The limitations include the following: incomplete characterization of the sequences that influence proteasomal degradation^52^ and insufficient cytosolic delivery into antigen-presenting cells. As a result, the degradation propensity for many immunogenic epitopes remained unclear, including for established disease models associated with cancer, viral, and bacterial epitopes.^53−55^

Among clinically studied vaccines, particularly personalized vaccines, only a small subset of the immunizing peptides demonstrated priming of cytotoxic T cells. To evaluate whether proteasomal degradation influences T cell priming in clinical settings, we used the model to evaluate degron activity across three clinical trial studies of personalized vaccine peptides: two for melanoma^56,57^ and one for glioblastoma.^58^ We evaluated the immunizing peptide sequences from these studies by dividing the results into two separate groups: presence (+) or absence (−) of CD8^+^ T cell responses after vaccination. The two groups were then plotted against the CDI values (left *Y* axis), in which the shading of the individual points reported the HLA-binding score (right *Y* axis).

Several trends emerged from these retrospective studies (Figure S29). From Ott and co-workers (2017),^56^ sequences exhibiting low CDI showed CD8^+^ T cell activation, indicating that proteasomal degradation of peptide-based vaccines favors T cell activation (Figure S29A); from Sahin and co-workers (2017),^57^ the analysis showed no difference between the unsuccessful sequences, which suggests that some RNA-encoded epitope sequences can be degraded before completion of ribosomal synthesis (Figure S29B); from Hilf and co-workers (2019),^58^ the successful sequences did not show a difference in mean CDI values; however, this study comprised few immunizing sequences and limits the ability to draw conclusions (Figure S29C). Taken together, these three studies suggest that degron activity is an important feature for designing vaccine epitopes. Nonetheless, further studies are needed to correlate the influence of vaccine formulation on the antigen degradation in vivo.

## Conclusion

This work provides a conceptual framework for combining degron sequences with vaccine epitopes. Although degron sequences are complex, machine learning accommodates these patterns for predicting degradation propensity. Incorporating degradation propensity into vaccine epitope design was shown to enhance epitope immunogenicity without altering the epitope. Degron sequences also enabled tuning of proteasomal degradation across disease models, particularly for designing the flanking residues of epitope sequences associated with model antigens, tumor antigens, and personalized neoantigens.

Essential to this work was the use of the PA/LF_N_ delivery system. PA/LF_N_ facilitated translocation of epitope sequences into cells for evaluating degradation propensity. Further analysis showed that relative proteasomal stability correlates with immune activation activity. These studies show promise for future efforts to improve vaccine epitope designs against tumors and pathogens in whole animal models and clinical settings.

## Supporting Information Available

Materials, methods, analytical, and biological data (PDF)
