# Supplementary material for: Design of Cytotoxic T Cell Epitopes by Machine Learning of Human Degrons
Source: ACS Cent Sci. 2024 Mar 6;10(4):793–802. doi: 10.1021/acscentsci.3c01544 (PMC11046456; doi:10.1021/acscentsci.3c01544)
Supplement: Supplementary file 1 — oc3c01544_si_001.pdf [file oc3c01544_si_001.pdf]

Supporting Information for

Design of Cytotoxic T Cell Epitopes by Machine Learning of Human Degrons

Nicholas L. Truex,[1,2†] Somesh Mohapatra,[3,4†] Mariane Melo,[5,6] Jacob Rodriguez,[1] Na Li,[5] Wuhbet Abraham,[5] Deborah Sementa,[1] Faycal Touti,[1] Derin B. Keskin,[7–12] Catherine J. Wu,[7–9,13] Darrell J. Irvine,[3,5,6,14,15] Rafael Gómez-Bombarelli,[3*] and Bradley L. Pentelute[1,5,9,16*]

[1] Department of Chemistry, Massachusetts Institute of Technology, Cambridge, MA 02139, USA.

[2] Department of Chemistry and Biochemistry, University of South Carolina, Columbia, SC 29208, USA.

[3] Department of Materials Science and Engineering, Massachusetts Institute of Technology, Cambridge, MA 02139, USA.

[4] Machine Intelligence and Manufacturing Operations Group, Massachusetts Institute of Technology, Cambridge, MA 02139, USA.

[5] The Koch Institute for Integrative Cancer Research, Massachusetts Institute of Technology, Cambridge, MA 02142, USA.

[6] Ragon Institute of Massachusetts General Hospital, Massachusetts Institute of Technology, and Harvard University, Cambridge, MA 02139, USA.

[7] Department of Medical Oncology, Dana-Farber Cancer Institute, Boston, MA 02215, USA.

[8] Harvard Medical School, Boston, MA 02215, USA.

[9] Broad Institute of MIT and Harvard, Cambridge, MA 02142, USA.

[10] Translational Immunogenomics Laboratory (TIGL), Dana-Farber Cancer Institute, Boston, MA 02215, USA.

[11] Department of Computer Science, Metropolitan College, Boston University, Boston, MA 02215, USA.

[12] Section for Bioinformatics, Department of Health Technology, Technical University of Denmark, Lyngby DK-2800, Denmark.

[13] Department of Medicine, Brigham and Women's Hospital, Boston, MA 02215, USA.

[14] Department of Biological Engineering, Massachusetts Institute of Technology, Cambridge, MA 02139, USA.

[15] Howard Hughes Medical Institute, Chevy Chase, MD 20815, USA.

[16] Center for Environmental Health Sciences, Massachusetts Institute of Technology, Cambridge, MA 02139, USA.

*Corresponding authors. Email: rafagb@mit.edu; blp@mit.edu
† These authors contributed equally to this work.

I. SUPPORTING FIGURES AND TABLES

**Table S1.**	Global property analysis of OVA peptides **1–12** and **1G–8G**.	S4
**Fig. S1.**	LC-MS analysis of LFN-OVA **1**.	S5
**Fig. S2.**	LC-MS analysis of LFN-OVA **2**.	S6
**Fig. S3.**	LC-MS analysis of LFN-OVA **3**.	S7
**Fig. S4.**	LC-MS analysis of LFN-OVA **4**.	S8
**Fig. S5.**	LC-MS analysis of LFN-OVA **5**.	S9
**Fig. S6.**	LC-MS analysis of LFN-OVA **6**.	S10
**Fig. S7.**	LC-MS analysis of LFN-OVA **7**.	S11
**Fig. S8.**	LC-MS analysis of LFN-OVA **8**.	S12
**Fig. S9.**	LC-MS analysis of LFN-OVA **9**.	S13
**Fig. S10.**	LC-MS analysis of LFN-OVA **10**.	S14
**Fig. S11.**	LC-MS analysis of LFN-OVA **11**.	S15
**Fig. S12.**	LC-MS analysis of LFN-OVA **12**.	S16
**Fig. S13.**	LC-MS analysis of LFN-OVA **1GG**.	S17
**Fig. S14.**	LC-MS analysis of LFN-OVA **2GG**.	S18
**Fig. S15.**	LC-MS analysis of LFN-OVA **3GG**.	S19
**Fig. S16.**	LC-MS analysis of LFN-OVA **4GG**.	S20
**Fig. S17.**	LC-MS analysis of LFN-OVA **5GG**.	S21
**Fig. S18.**	LC-MS analysis of LFN-OVA **6GG**.	S22
**Fig. S19.**	LC-MS analysis of LFN-OVA **7GG**.	S23
**Fig. S20.**	LC-MS analysis of LFN-OVA **8GG**.	S24
**Fig. S21.**	Flow cytometry analysis of BV421-labeled Thy1.1+OT-1 cells after 24 h.	S25
**Fig. S22.**	Flow cytometry analysis of BV421-labeled Thy1.1+OT-1 cells after 72 h.	S25
**Fig. S23.**	Average CDI Heatmaps.	S26
**Fig. S24.**	Sequence logo plots.	S26
**Table S2.**	Global property analysis of $\mathbf{CDI_{LO}}$ and $\mathbf{CDI_{HI}}$ peptides.	S26
**Fig. S25.**	LC-MS analysis of LFN-OVA $\mathbf{CDI_{LO}}$.	S27
**Fig. S26.**	LC-MS analysis of LFN-OVA $\mathbf{CDI_{HI}}$.	S28
**Fig. S27.**	LC-MS analysis of LFN-PMEL $\mathbf{CDI_{LO}}$.	S29
**Fig. S28.**	LC-MS analysis of LFN-PMEL $\mathbf{CDI_{HI}}$.	S30
**Fig. S29.**	Retrospective analysis of clinically studied vaccine sequences.	S31

II. Materials and Methods

Materials	S32
General Equation	S32
Synthesis and purification of antigen peptides	S33
Protein expression and purification	S34
Sortase-mediated ligations	S35
LC-MS protein characterization	S36
Western blot	S36
Endotoxin testing and removal	S37
Murine immune response	S37
Mixed lymphocyte reaction	S38
Statistical analysis	S39
Representation of peptides	S39
Machine learning	S39
Genetic algorithm for generation of new peptides	S39
Selection of peptides for experimental evaluation	S40

I. SUPPORTING FIGURES AND TABLES

Table S1. Global property analysis of OVA **1–12** and **1G–8G** peptides.

Peptide	Molecular Weight (g/mol)	Aromaticity	Isoelectric Point	Charge at pH 7	GRAVY	Secondary Structure - Helix Fraction	Secondary Structure - Turn Fraction	Secondary Structure - Sheet Fraction
OVA 1	1747.0	0.062	4.25	-2.23	-0.01	0.44	0.31	0.38
OVA 2	1848.1	0.059	4.25	-2.23	-0.05	0.41	0.29	0.41
OVA 3	1977.2	0.056	4.33	-3.22	-0.24	0.44	0.28	0.39
OVA 4	2163.4	0.105	4.09	-3.23	-0.28	0.42	0.26	0.42
OVA 5	2264.5	0.100	4.09	-3.23	-0.30	0.40	0.25	0.45
OVA 6	2351.6	0.095	4.09	-3.23	-0.32	0.38	0.29	0.43
OVA 7	2438.6	0.091	4.09	-3.23	-0.35	0.36	0.32	0.41
OVA 8	3567.0	0.065	4.77	-2.23	-0.64	0.42	0.26	0.36
OVA 9	2731.0	0.040	4.95	-1.24	-0.59	0.32	0.32	0.32
OVA 10	1978.1	0.056	4.09	-3.23	-0.65	0.39	0.33	0.33
OVA 11	2689.8	0.042	4.34	-6.13	-1.36	0.42	0.29	0.29
OVA 12	3036.1	0.037	4.06	-6.22	-1.83	0.37	0.48	0.15
OVA 1G	1861.1	0.056	4.25	-2.23	-0.06	0.39	0.39	0.33
OVA 2G	1962.2	0.053	4.25	-2.23	-0.09	0.37	0.37	0.37
OVA 3G	2091.3	0.050	4.09	-3.23	-0.26	0.40	0.35	0.35
OVA 4G	2277.5	0.095	4.09	-3.23	-0.29	0.38	0.33	0.38
OVA 5G	2378.6	0.091	4.09	-3.23	-0.31	0.36	0.32	0.41
OVA 6G	2465.6	0.087	4.09	-3.23	-0.33	0.35	0.35	0.39
OVA 7G	2552.7	0.083	4.09	-3.23	-0.35	0.33	0.38	0.38
OVA 8G	3681.1	0.061	4.77	-2.23	-0.62	0.39	0.30	0.33

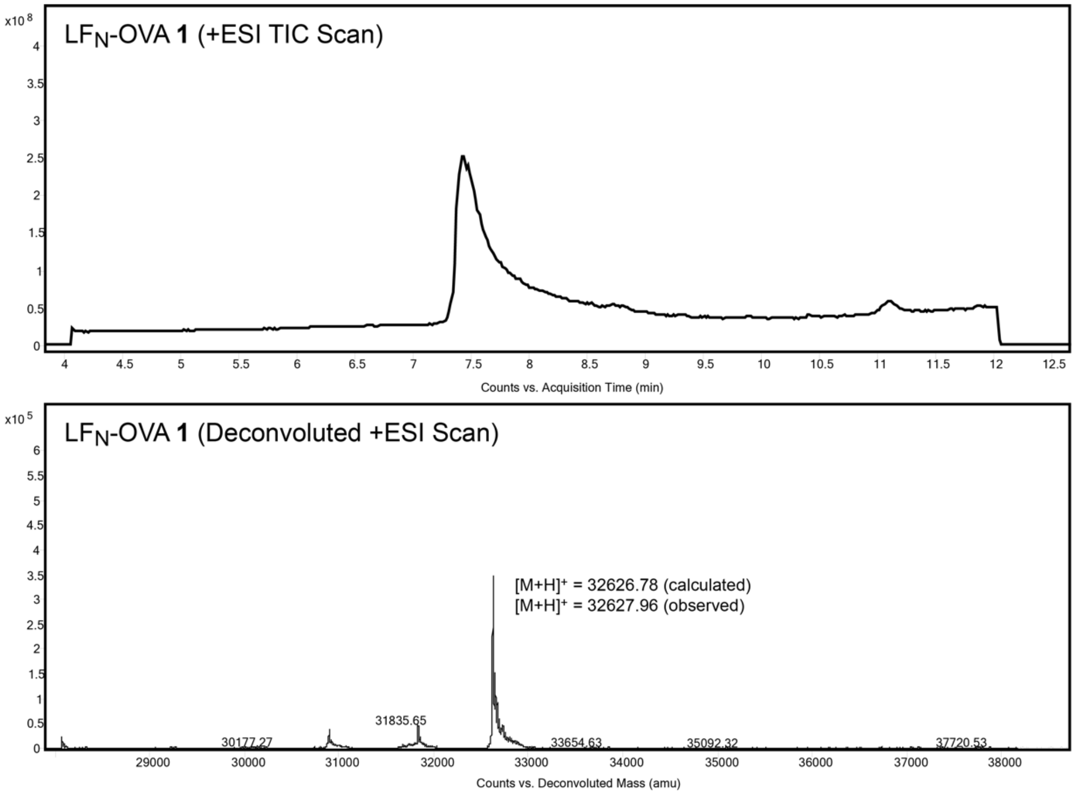

Fig. S1. LC-MS analysis of LF_N-OVA **1**. Deconvoluted spectrum is shown from a portion of the spectral window from +ESI TIC Scan (7.34–7.89 min).

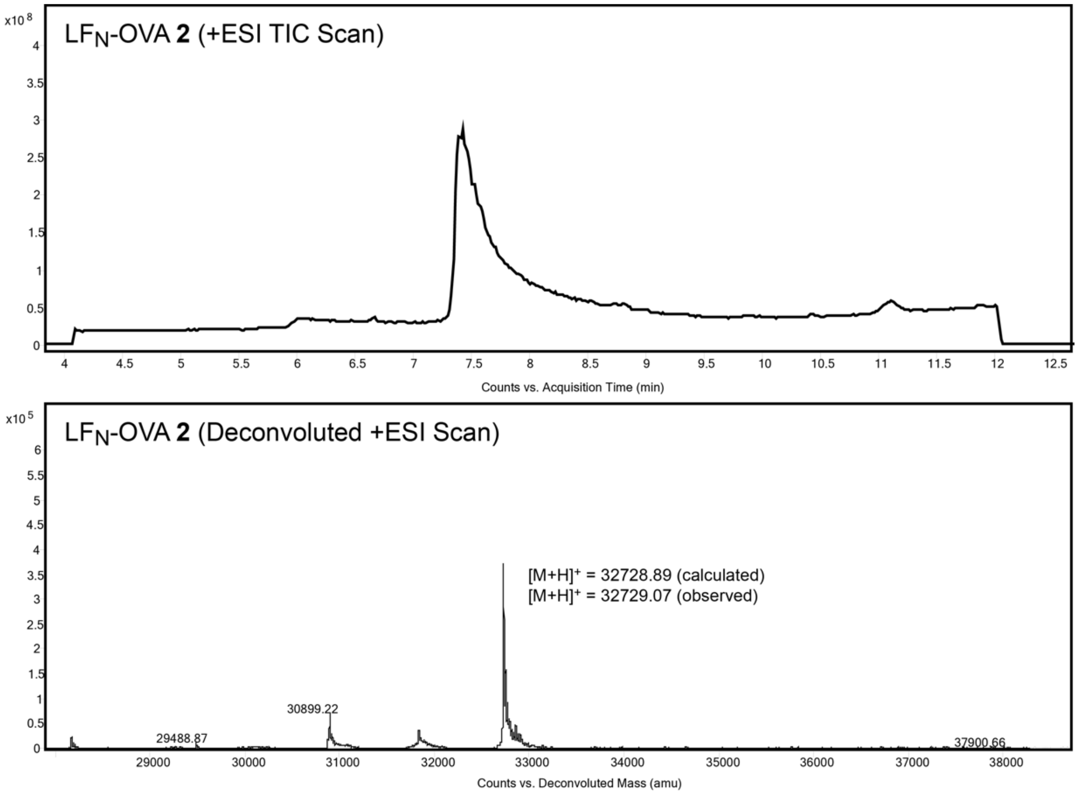

Fig. S2. LC-MS analysis of LF_N-OVA **2**. Deconvoluted spectrum is shown from a portion of the spectral window from +ESI TIC Scan (7.32–7.92 min).

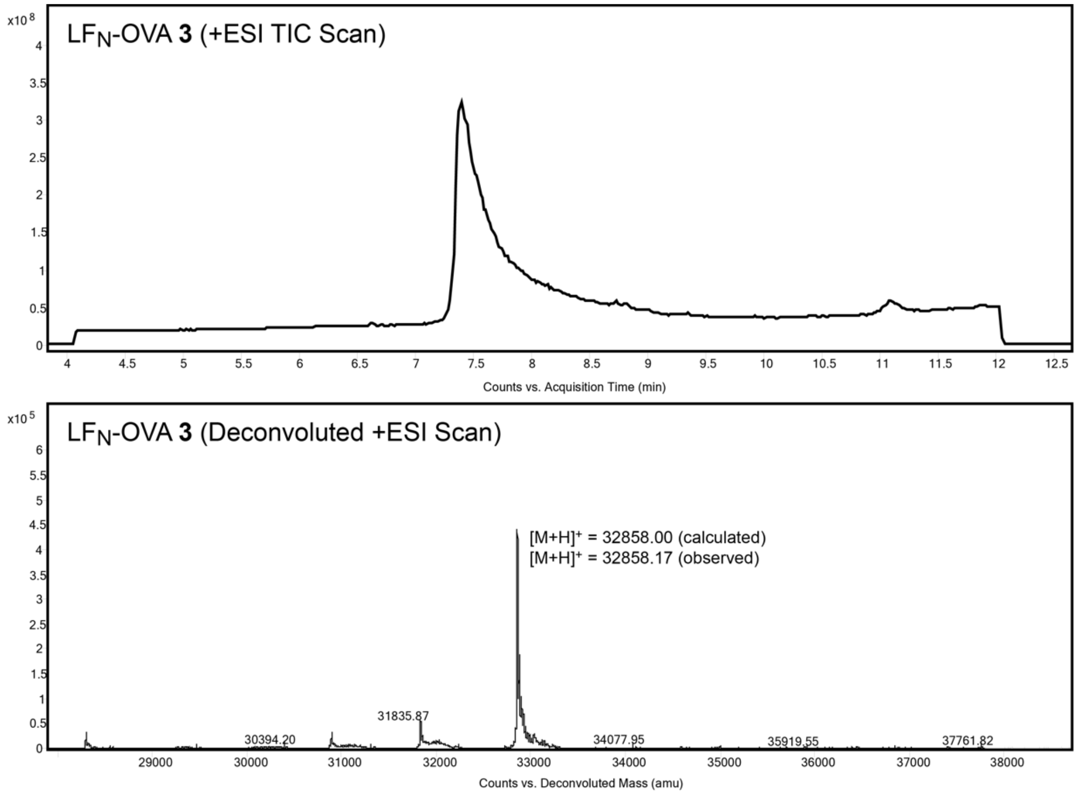

Fig. S3. LC-MS analysis of LF_N-OVA **3**. Deconvoluted spectrum is shown from a portion of the spectral window from +ESI TIC Scan (7.31–7.96 min).

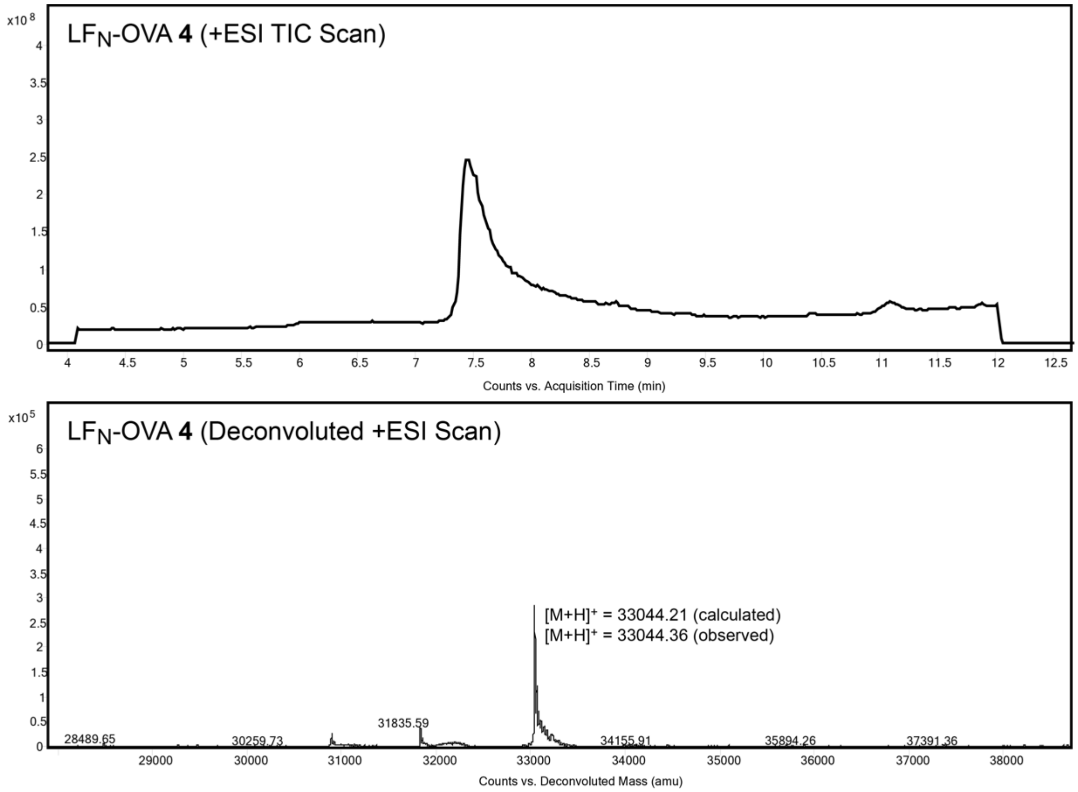

Fig. S4. LC-MS analysis of LF_N-OVA **4**. Deconvoluted spectrum is shown from a portion of the spectral window from +ESI TIC Scan (7.36–8.00 min).

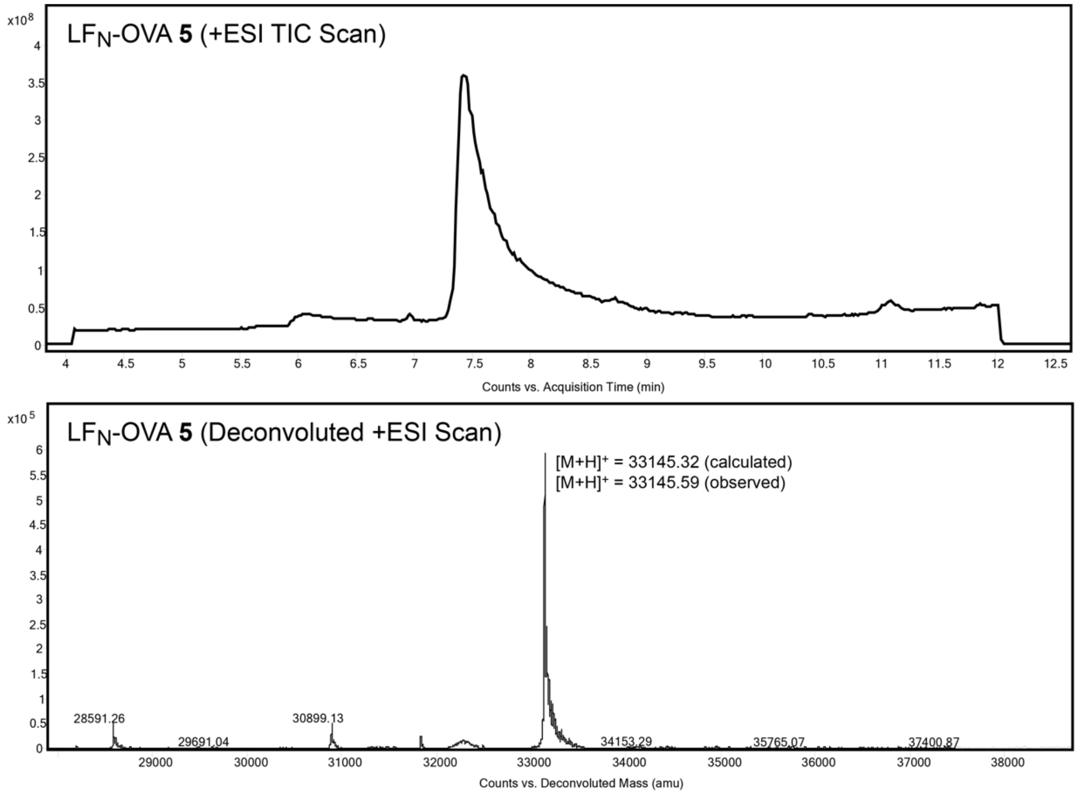

Fig. S5. LC-MS analysis of LF$_N$-OVA **5**. Deconvoluted spectrum is shown from a portion of the spectral window from +ESI TIC Scan (7.33–7.88 min).

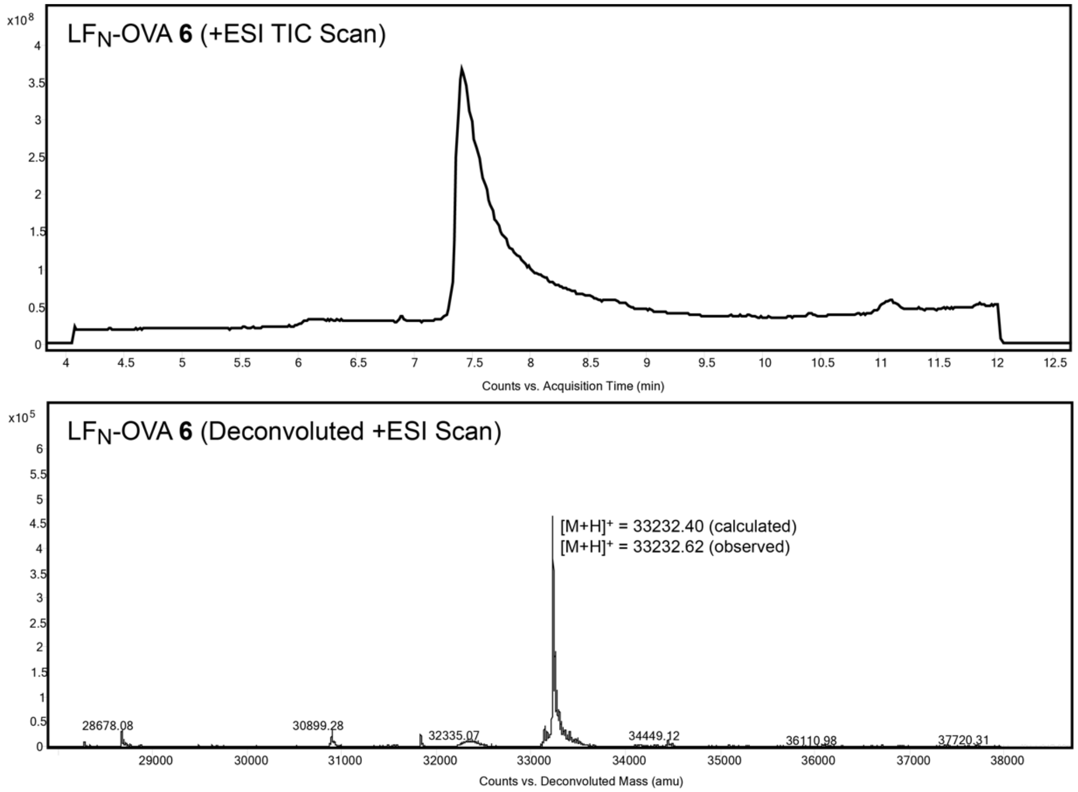

Fig. S6. LC-MS analysis of LF$_N$-OVA **6**. Deconvoluted spectrum is shown from a portion of the spectral window from +ESI TIC Scan (7.33–8.06 min).

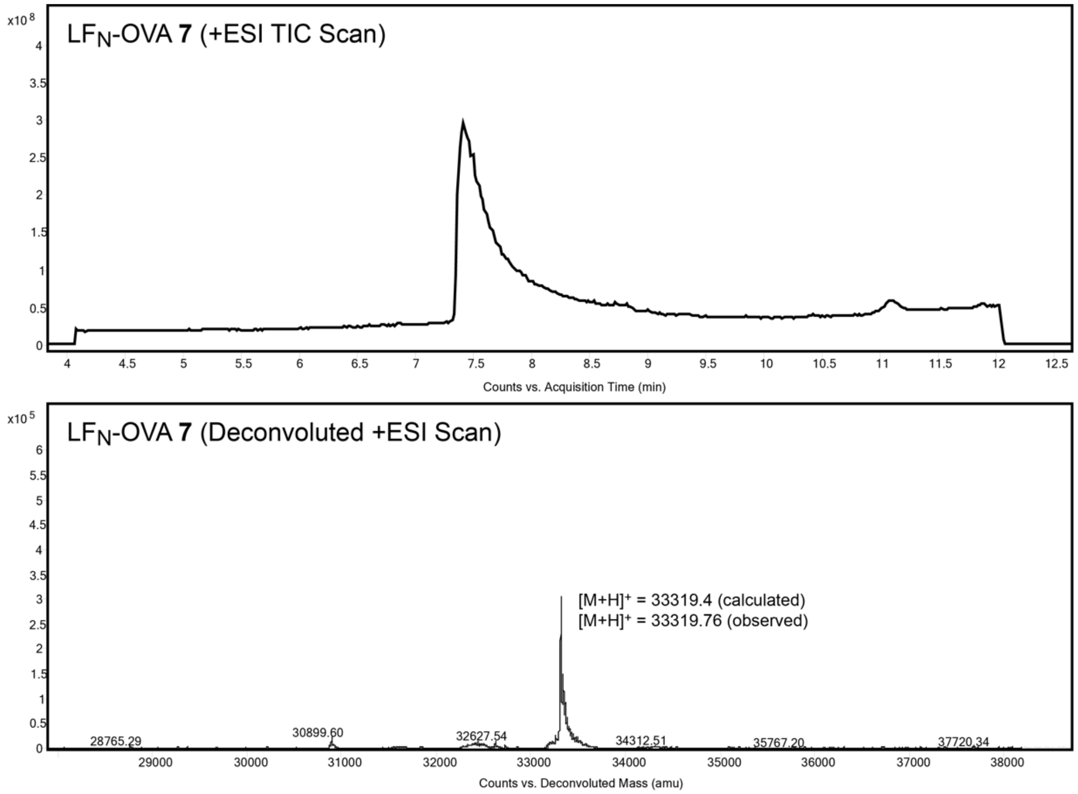

Fig. S7. LC-MS analysis of LF_N-OVA **7**. Deconvoluted spectrum is shown from a portion of the spectral window from +ESI TIC Scan (7.35–7.94 min).

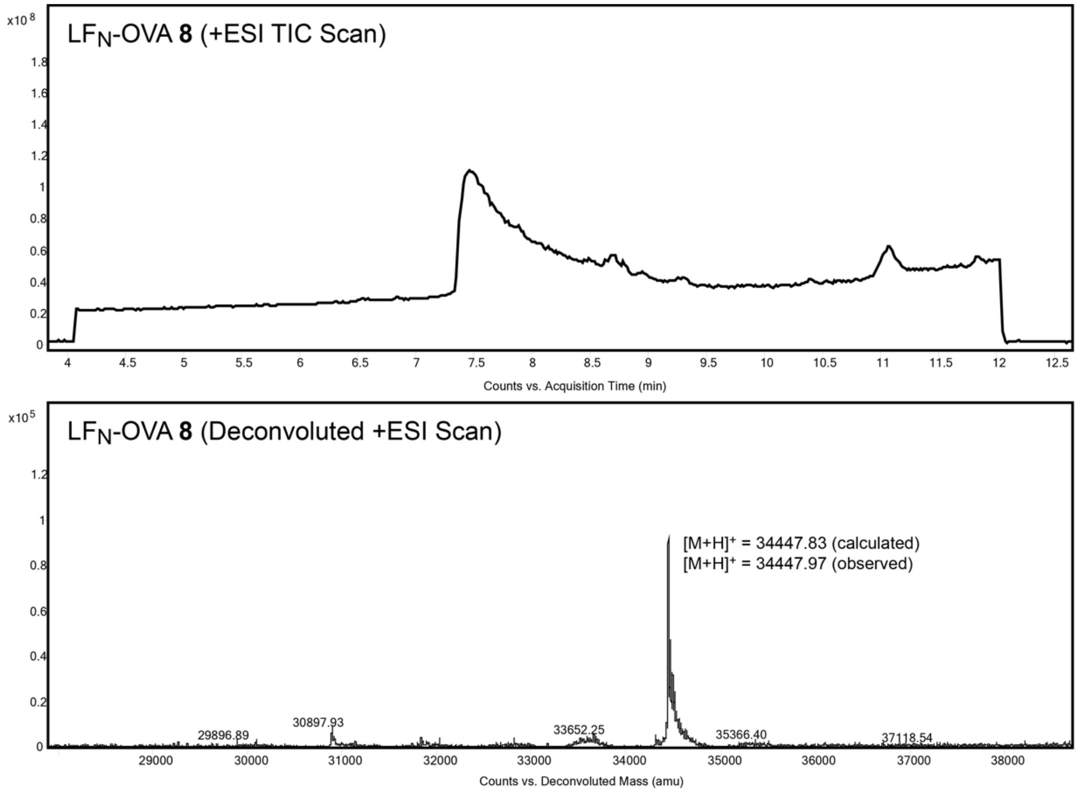

Fig. S8. LC-MS analysis of LF_N-OVA **8**. Deconvoluted spectrum is shown from a portion of the spectral window from +ESI TIC Scan (7.35–8.14 min).

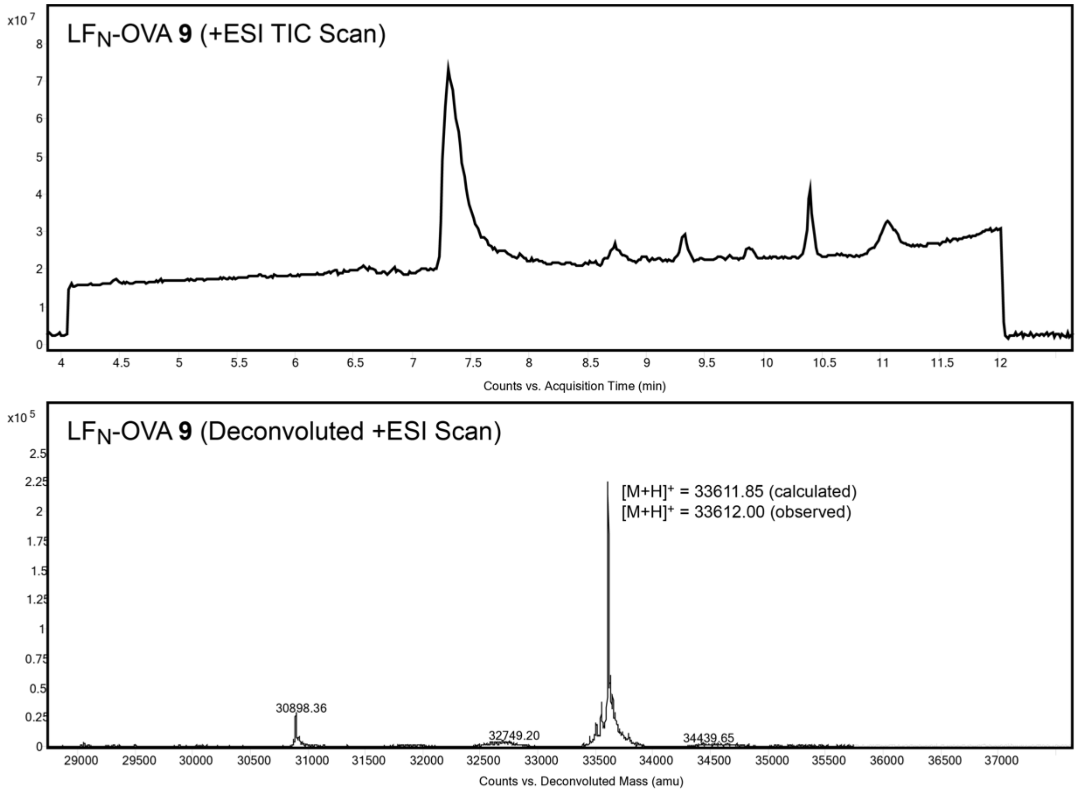

Fig. S9. LC-MS analysis of LF_N-OVA **9**. Deconvoluted spectrum is shown from a portion of the spectral window from +ESI TIC Scan (7.24–7.64 min).

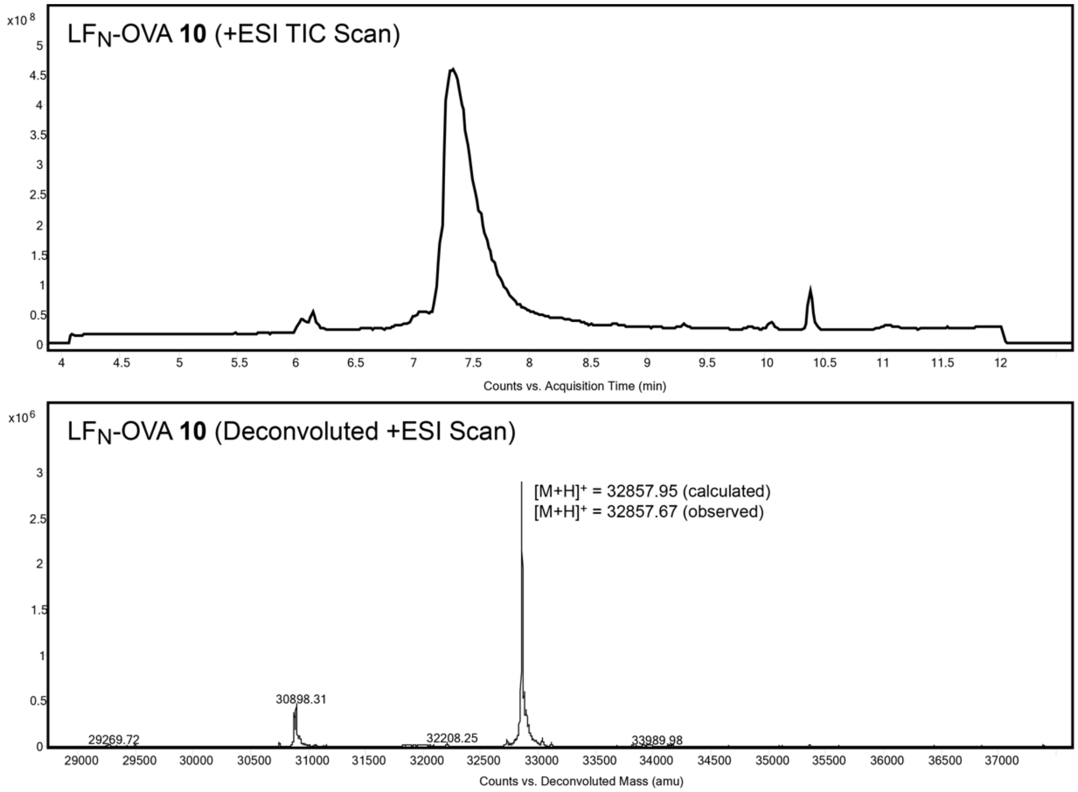

Fig. S10. LC-MS analysis of LF_N-OVA **10**. Deconvoluted spectrum is shown from a portion of the spectral window from +ESI TIC Scan (7.22–7.73 min).

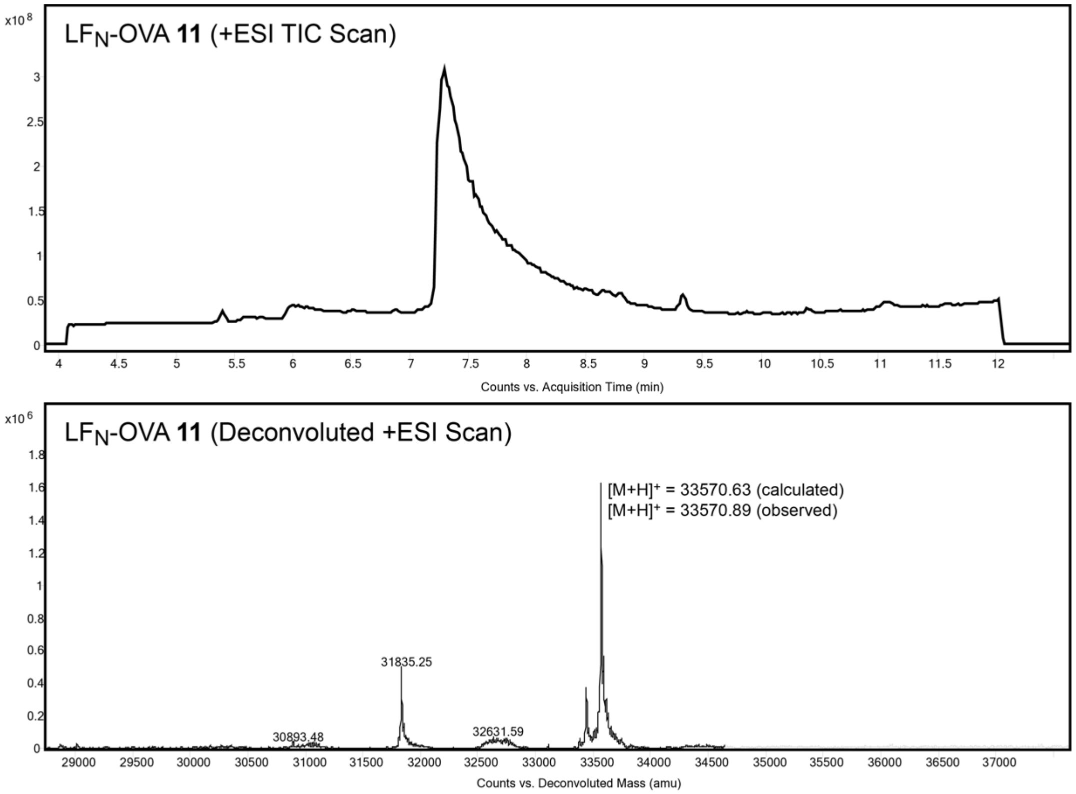

Fig. S11. LC-MS analysis of LF_N-OVA **11**. Deconvoluted spectrum is shown from a portion of the spectral window from +ESI TIC Scan (7.28 min).

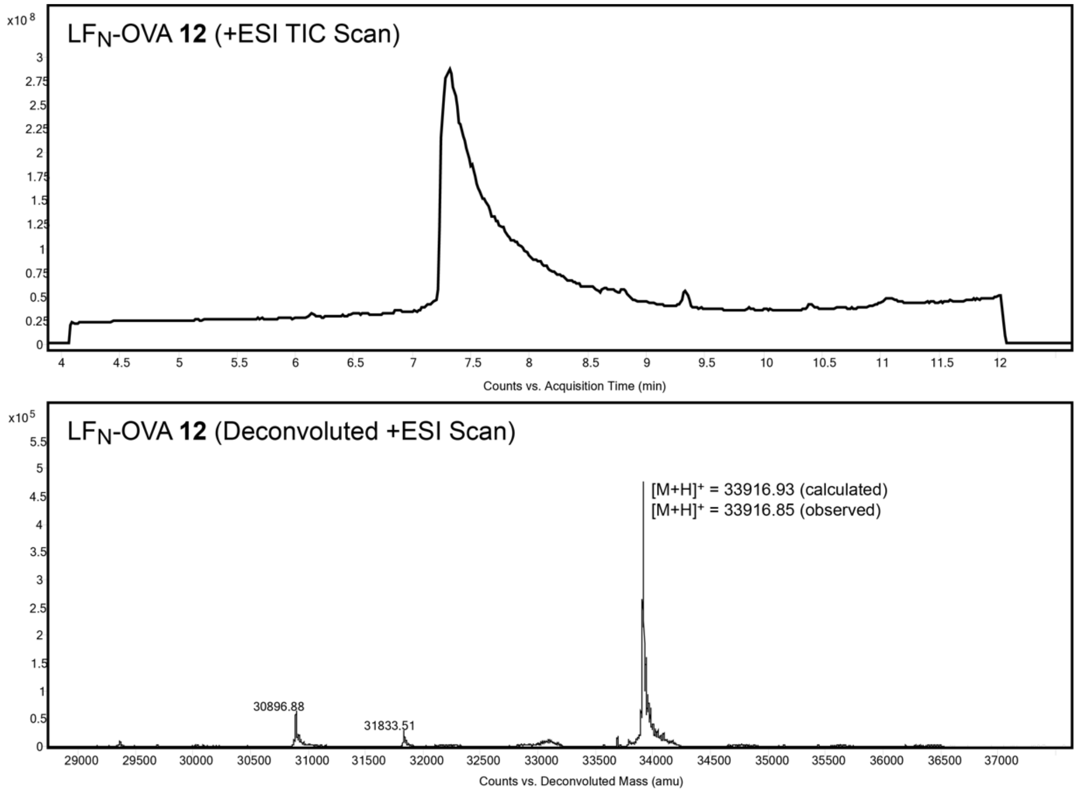

Fig. S12. LC-MS analysis of LF_N-OVA **12**. Deconvoluted spectrum is shown from a portion of the spectral window from +ESI TIC Scan (7.23–8.16 min).

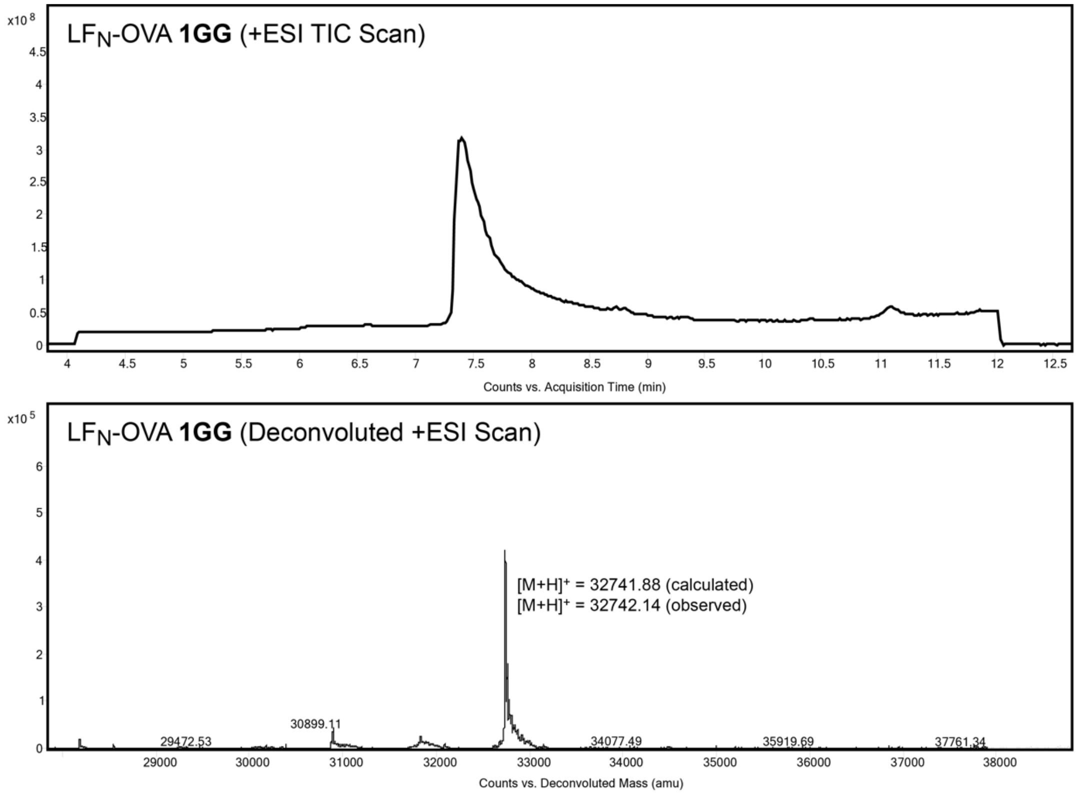

Fig. S13. LC-MS analysis of LF_N-OVA **1GG**. Deconvoluted spectrum is shown from a portion of the spectral window from +ESI TIC Scan (7.33–7.97 min).

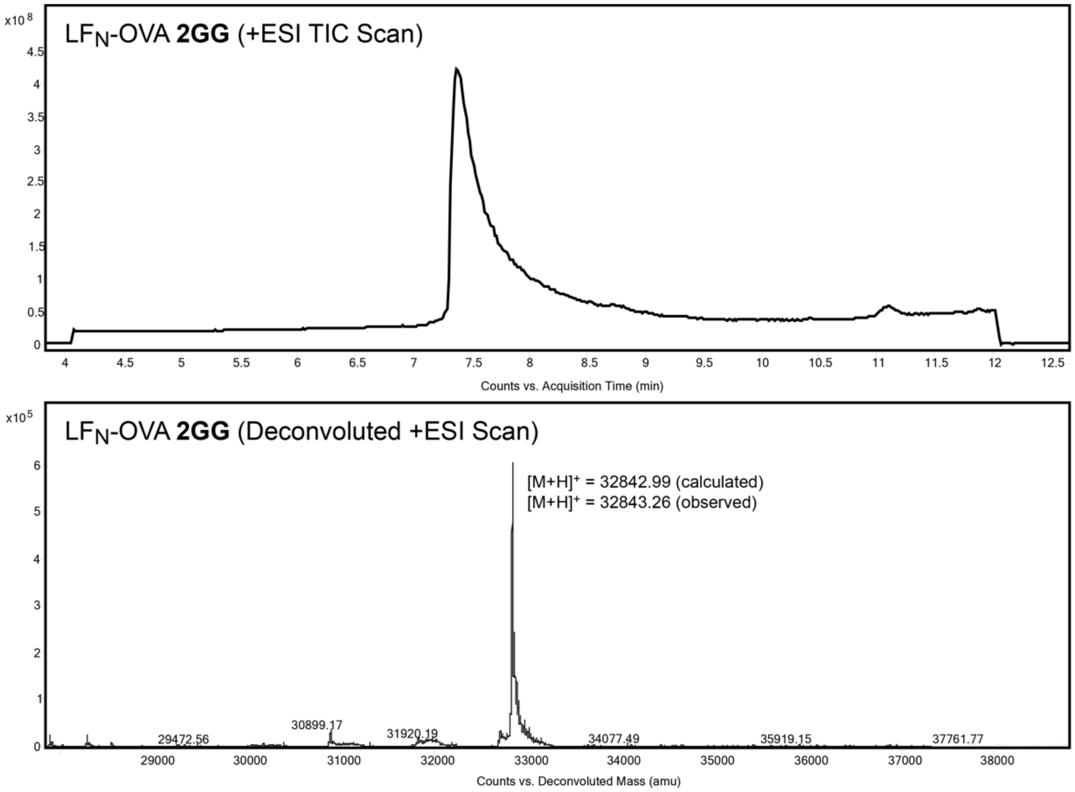

Fig. S14. LC-MS analysis of LF_N-OVA **2GG**. Deconvoluted spectrum is shown from a portion of the spectral window from +ESI TIC Scan (7.31–7.96 min).

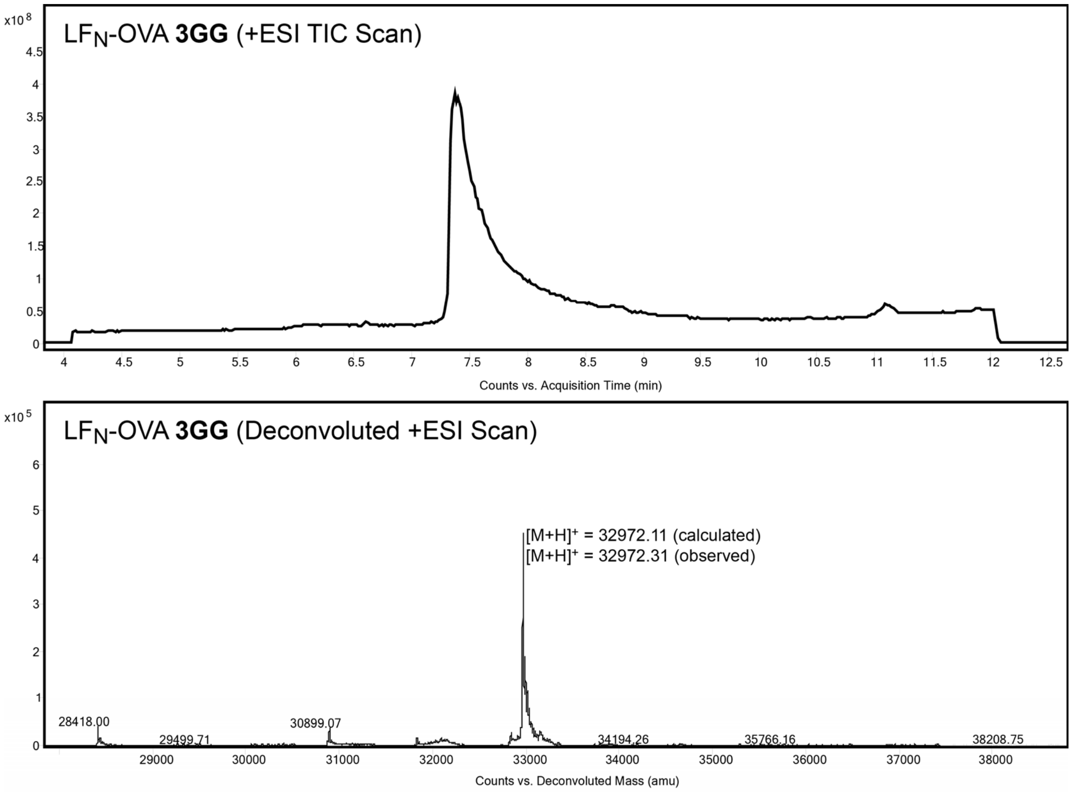

Fig. S15. LC-MS analysis of LF_N-OVA **3GG**. Deconvoluted spectrum is shown from a portion of the spectral window from +ESI TIC Scan (7.31–8.03 min).

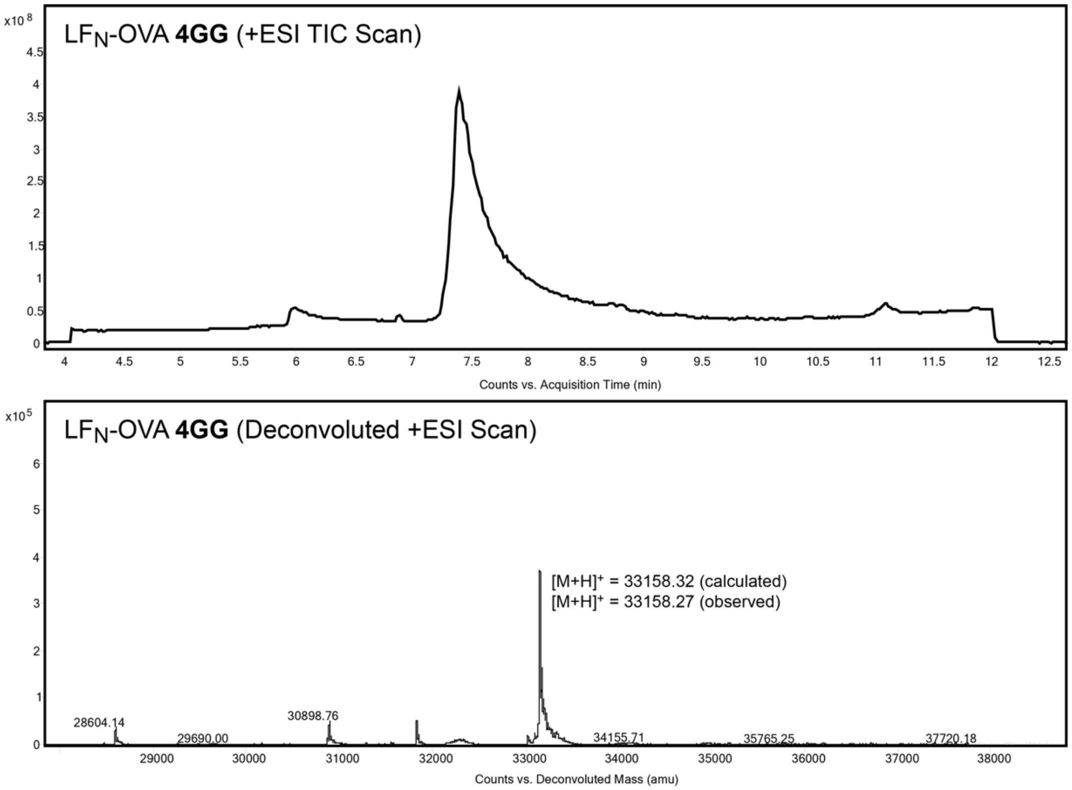

Fig. S16. LC-MS analysis of LF$_N$-OVA **4GG**. Deconvoluted spectrum is shown from a portion of the spectral window from +ESI TIC Scan (7.29–8.04 min).

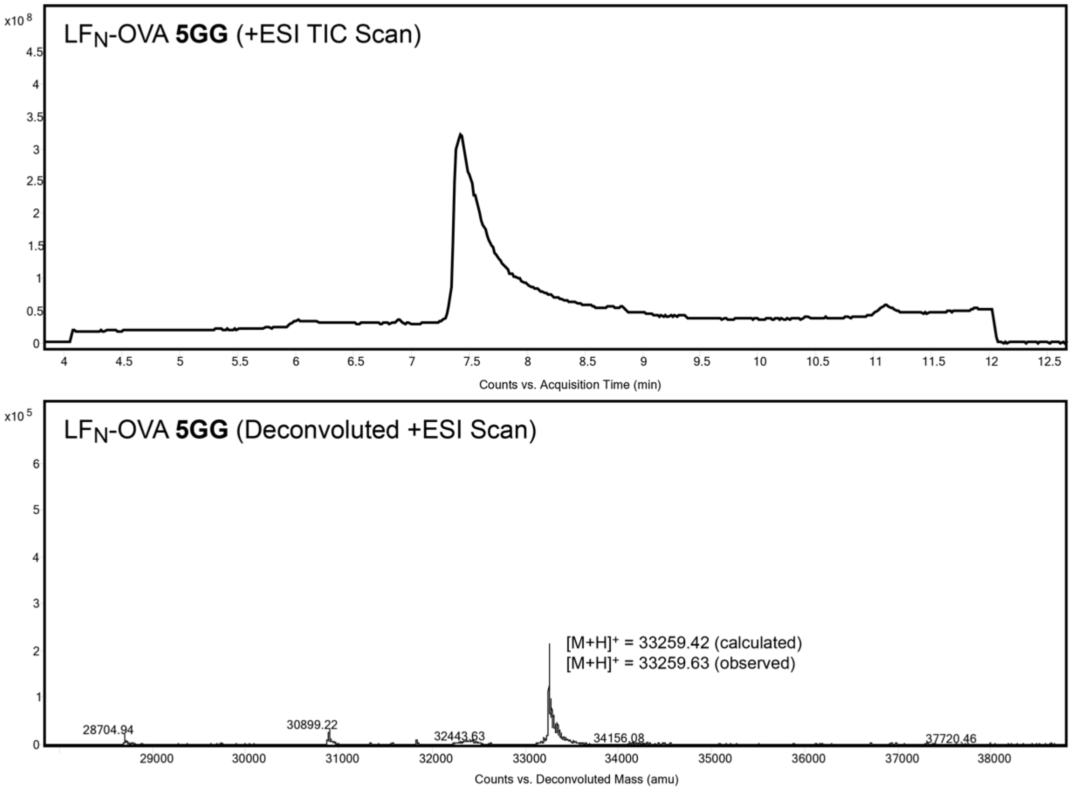

Fig. S17. LC-MS analysis of LF_N-OVA **5GG**. Deconvoluted spectrum is shown from a portion of the spectral window from +ESI TIC Scan (7.34–8.42 min).

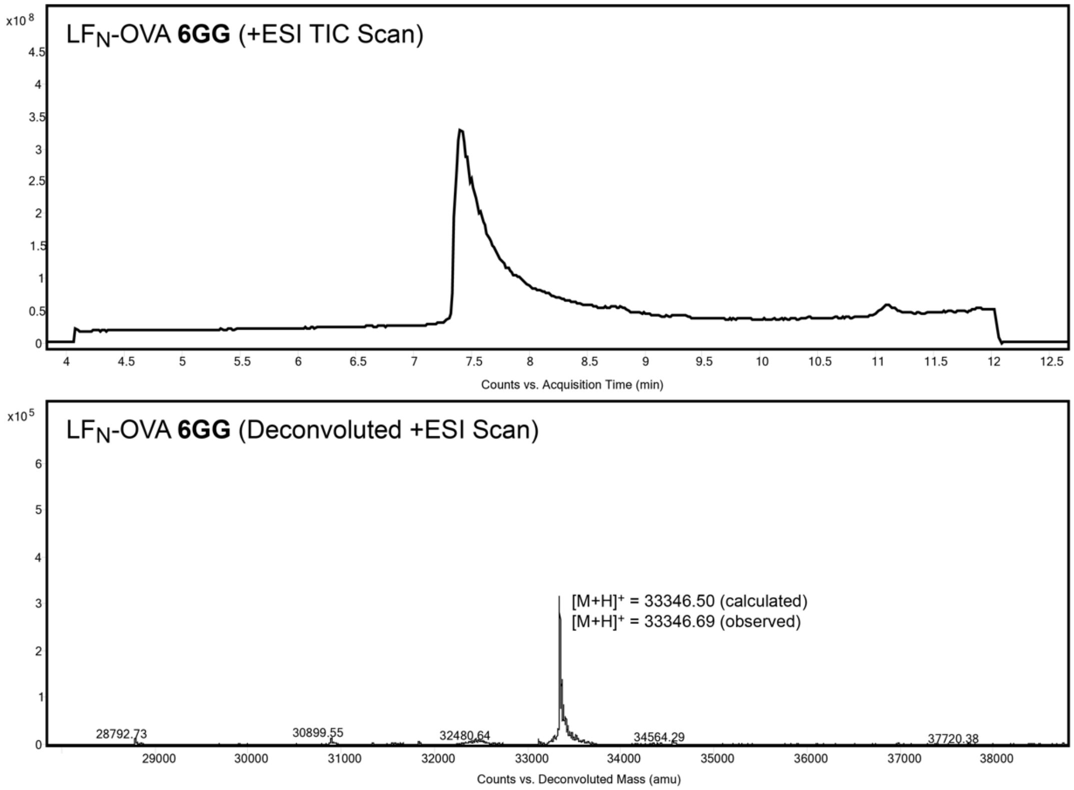

Fig. S18. LC-MS analysis of LF_N-OVA **6GG**. Deconvoluted spectrum is shown from a portion of the spectral window from +ESI TIC Scan (7.34–8.30 min.).

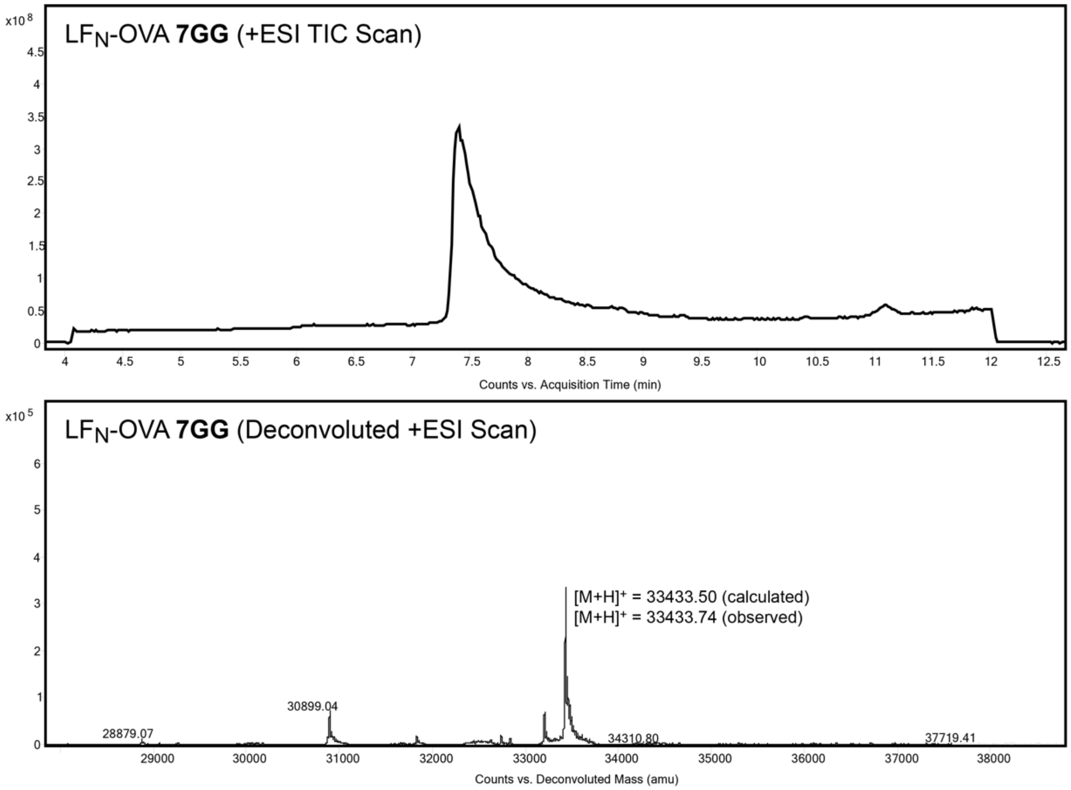

Fig. S19. LC-MS analysis of LF_N-OVA **7GG**. Deconvoluted spectrum is shown from a portion of the spectral window from +ESI TIC Scan (7.31–7.91 min.).

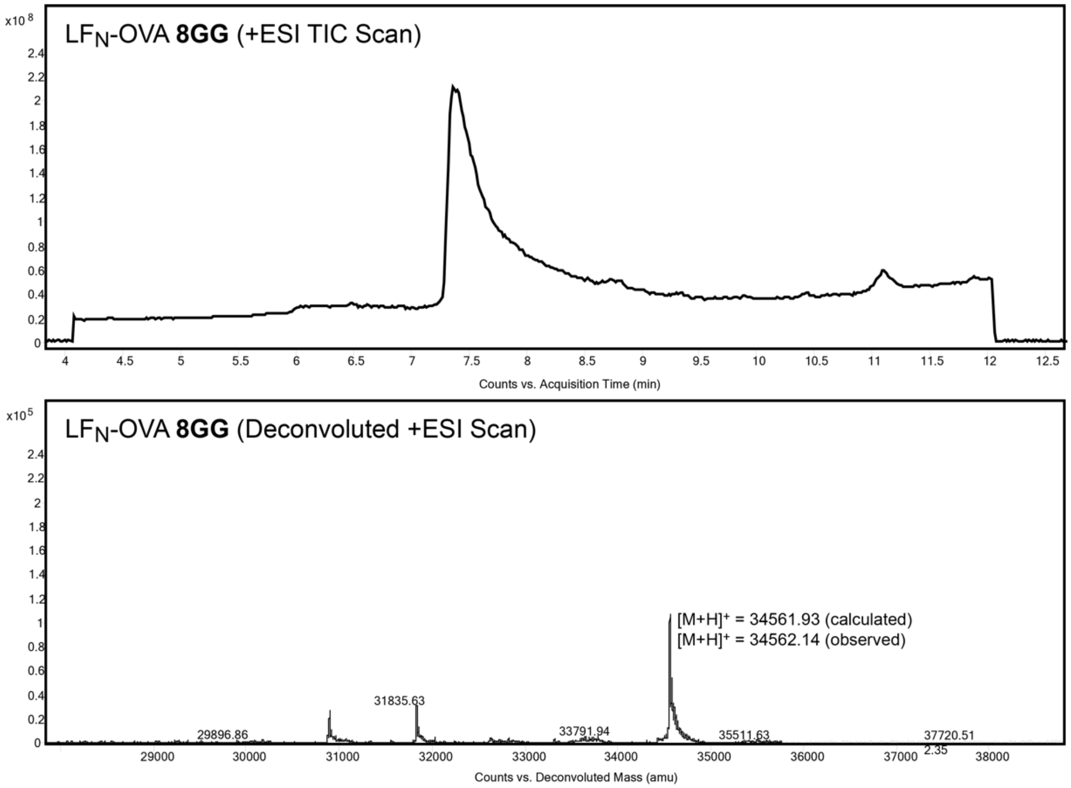

Fig. S20. LC-MS analysis of LF$_N$-OVA **8GG**. Deconvoluted spectrum is shown from a portion of the spectral window from +ESI TIC Scan (7.29–8.44 min.).

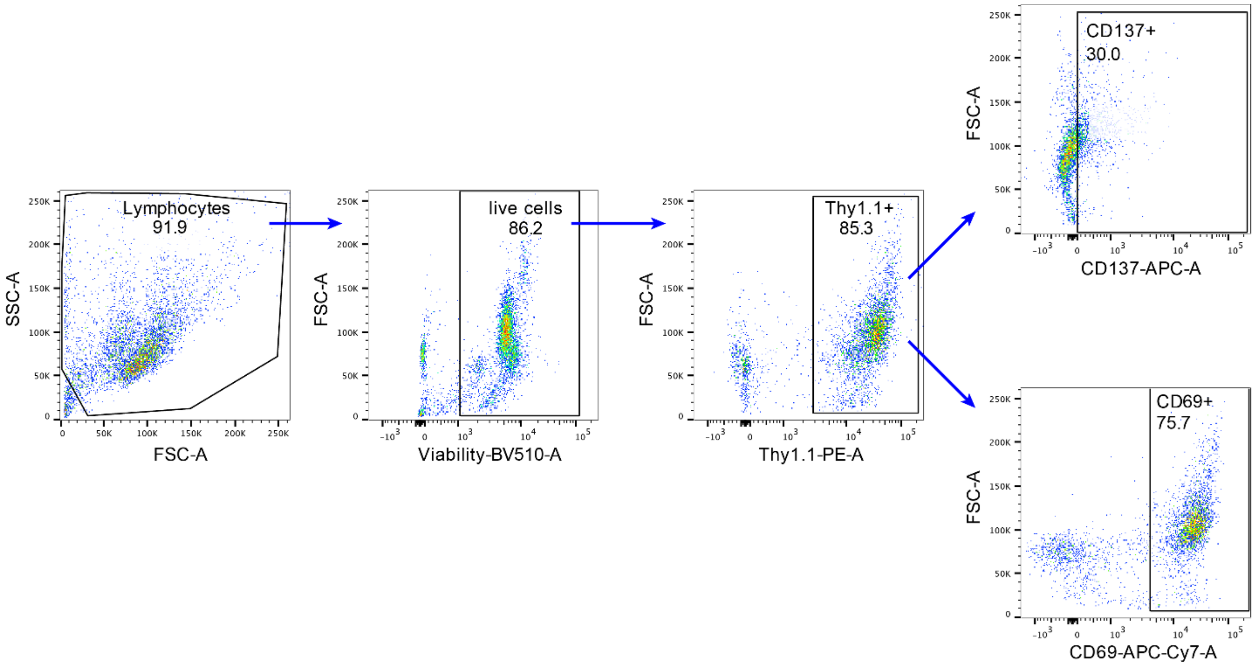

Fig. S21. Gating strategy for flow cytometry analysis of BV421-labeled Thy1.1^{+}OT-1 lymphocytes after 24 h incubation with murine DCs containing translocated OVA constructs. Data are representative of at least three independent experiments.

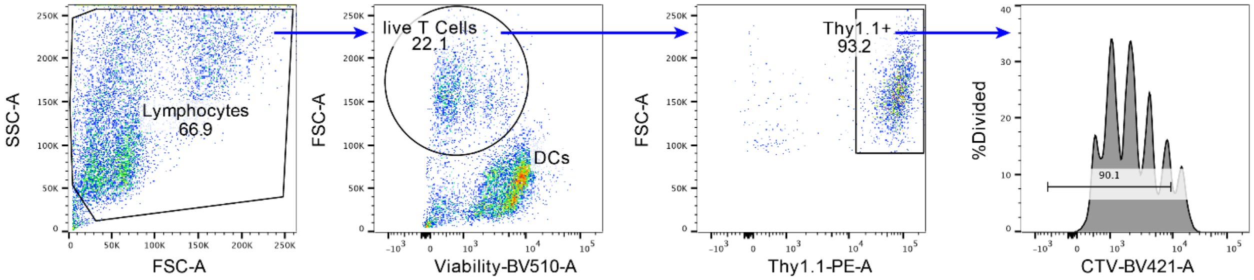

Fig. S22. Gating strategy for flow cytometry analysis of BV421-labeled Thy1.1^{+}OT-1 lymphocytes after 72 h incubation with DCs containing translocated OVA constructs. Data are representative of at least three independent experiments.

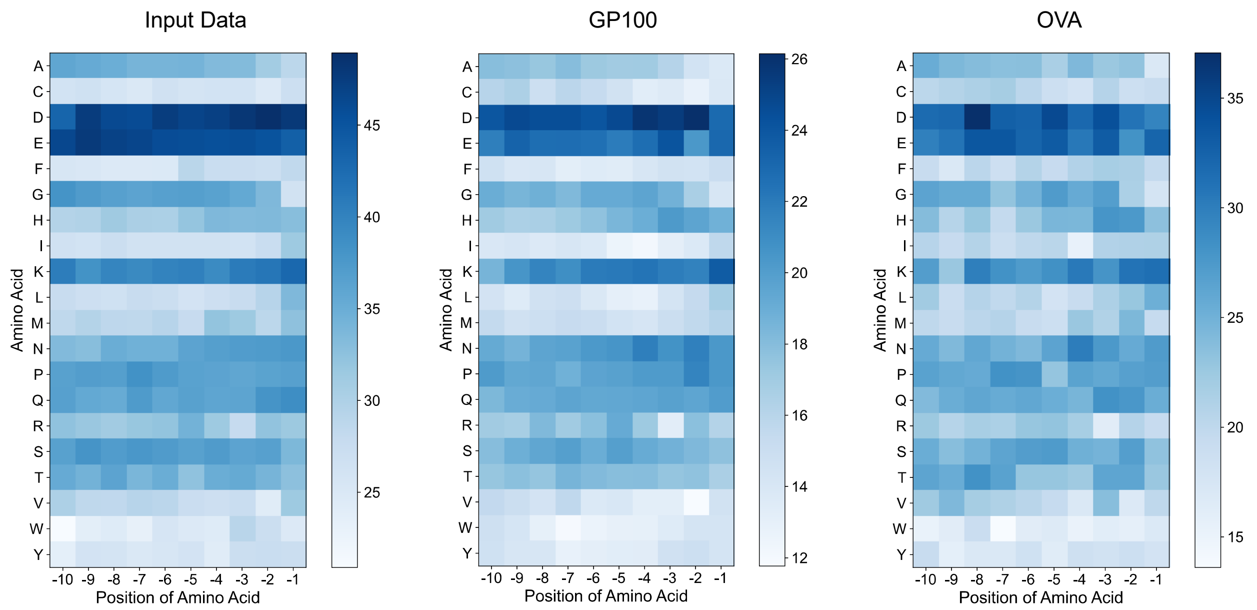

Fig. S23. Average CDI Heatmap for all sequences in the training data, and sequences generated during the genetic algorithm optimization for gp100 and OVA. Only the last 10 residues at the C-terminus have been used to plot the heatmap.

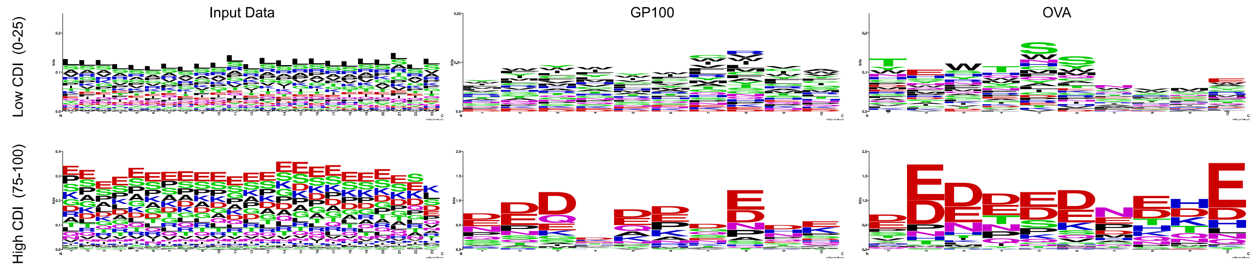

Fig. S24. Sequence logos for sequences in the training data, and sequences generated during the genetic algorithm optimization for gp100 and OVA. The plots are separated by their CDI scores – low (0-25) and high (75-100).

Table S2. Global property analysis of $\mathbf{CDI_{LO}}$ and $\mathbf{CDI_{HI}}$ peptides.

Peptide	Molecular Weight	Aromaticity	Isoelectric Point	Charge at pH 7	GRAVY	Secondary Structure - Helix Fraction	Secondary Structure - Turn Fraction	Secondary Structure - Sheet Fraction
OVA $\mathbf{CDI_{LO}}$	2416.6	0.087	4.25	-2.23	-0.16	0.35	0.44	0.30
OVA $\mathbf{CDI_{HI}}$	2815.0	0.043	4.47	-5.96	-1.36	0.39	0.26	0.26
gp100 $\mathbf{CDI_{LO}}$	2530.9	0.042	8.79	0.80	-0.30	0.29	0.42	0.29
gp100 $\mathbf{CDI_{HI}}$	2712.1	0.042	8.55	0.97	-1.26	0.38	0.38	0.17

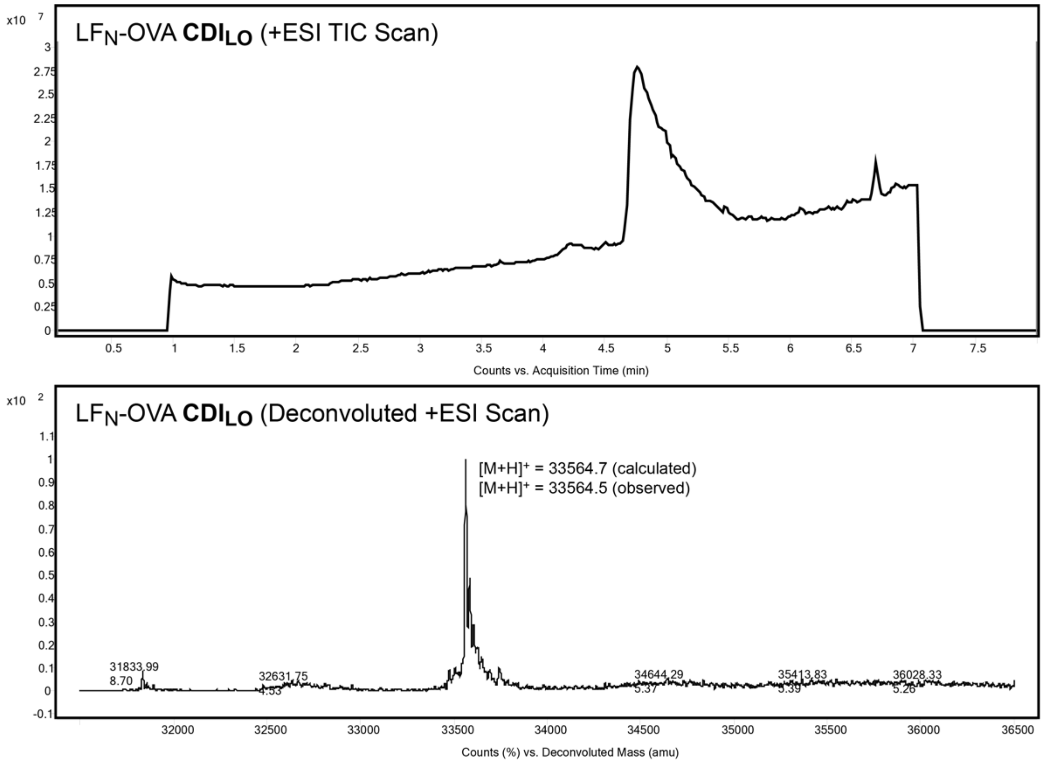

Fig. S25. LC-MS analysis of LF$_N$-OVA **CDI$_{LO}$**. Deconvoluted spectrum is shown from a portion of the spectral window from +ESI TIC Scan (4.68–5.49 min).

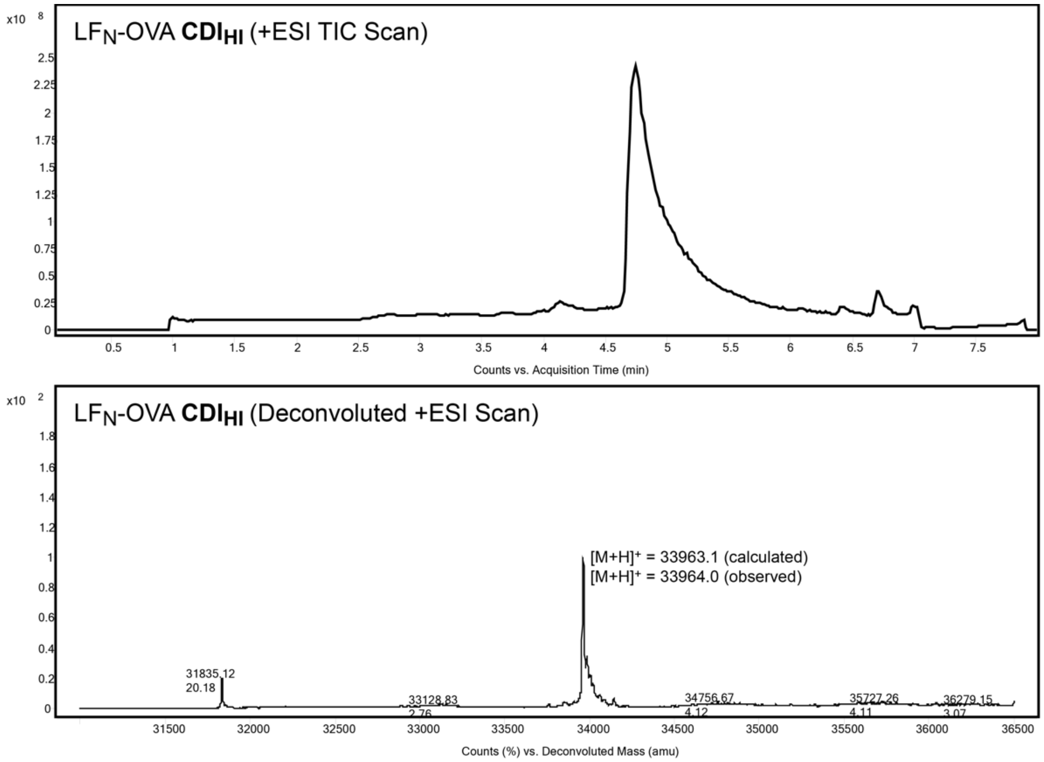

Fig. S26. LC-MS analysis of LF$_N$-OVA **CDI$_{HI}$**. Deconvoluted spectrum is shown from a portion of the spectral window from +ESI TIC Scan (4.93–5.28 min).

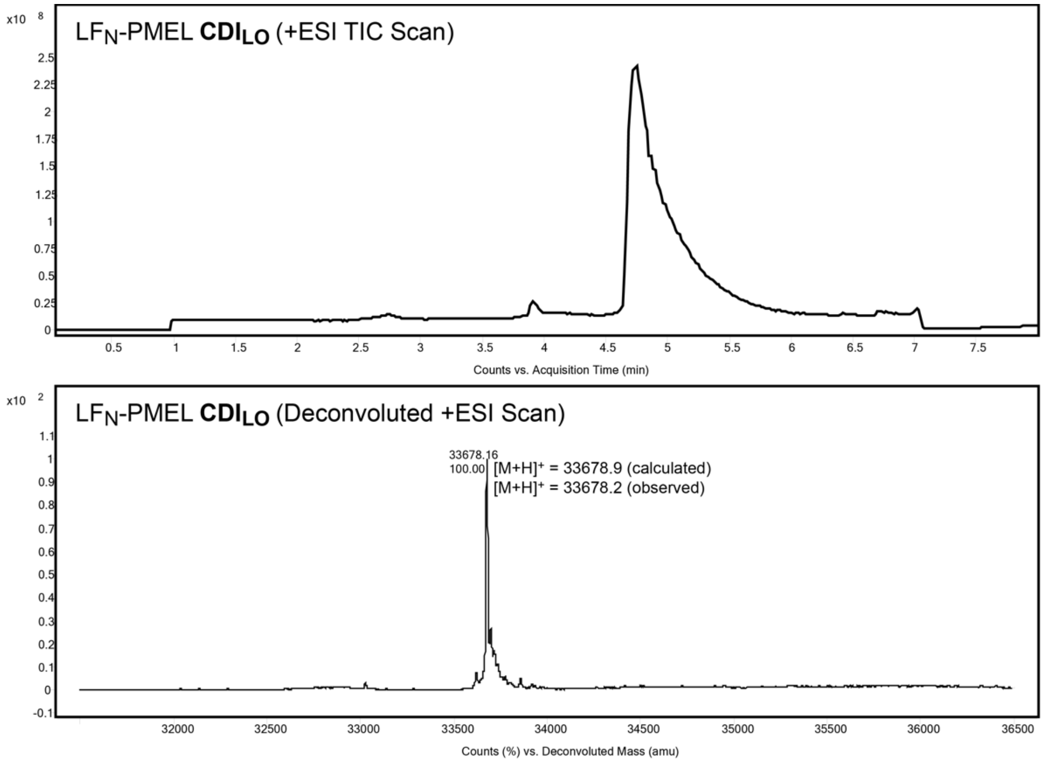

Fig. S27. LC-MS analysis of LF_N-PMEL $\mathbf{CDI_{LO}}$. Deconvoluted spectrum is shown from a portion of the spectral window from +ESI TIC Scan (4.65–5.35 min).

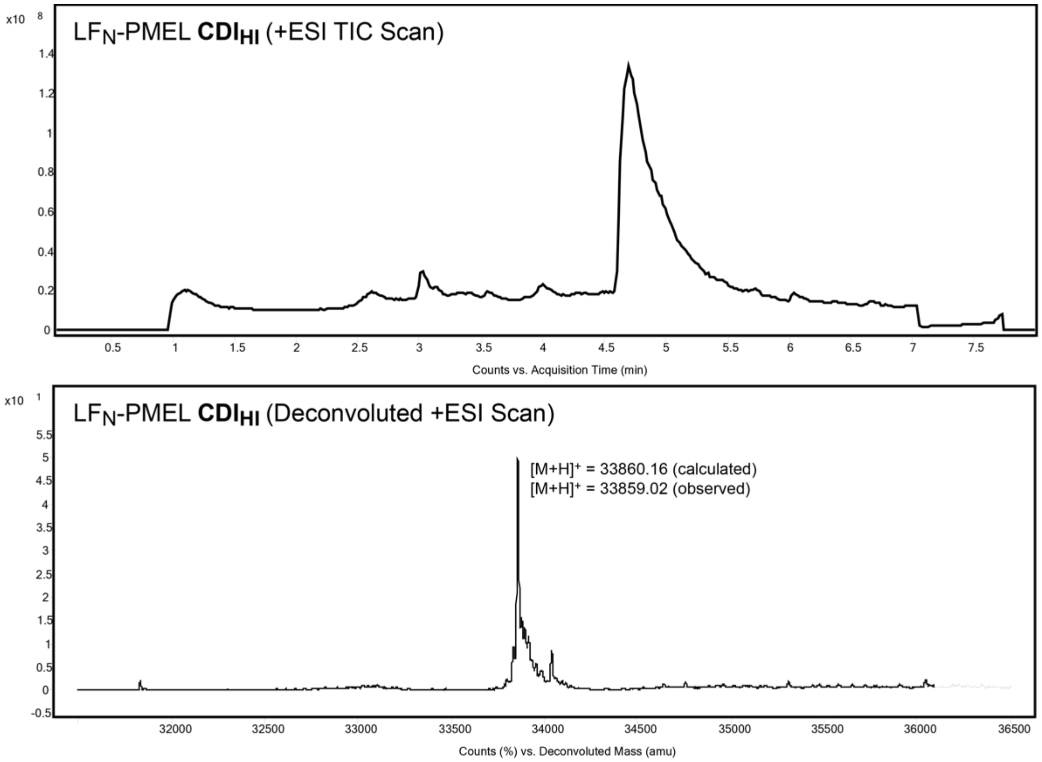

Fig. S28. LC-MS analysis of LF$_N$-PMEL **CDI$_{HI}$**. Deconvoluted spectrum is shown from a portion of the spectral window from +ESI TIC Scan (4.61–5.16 min).

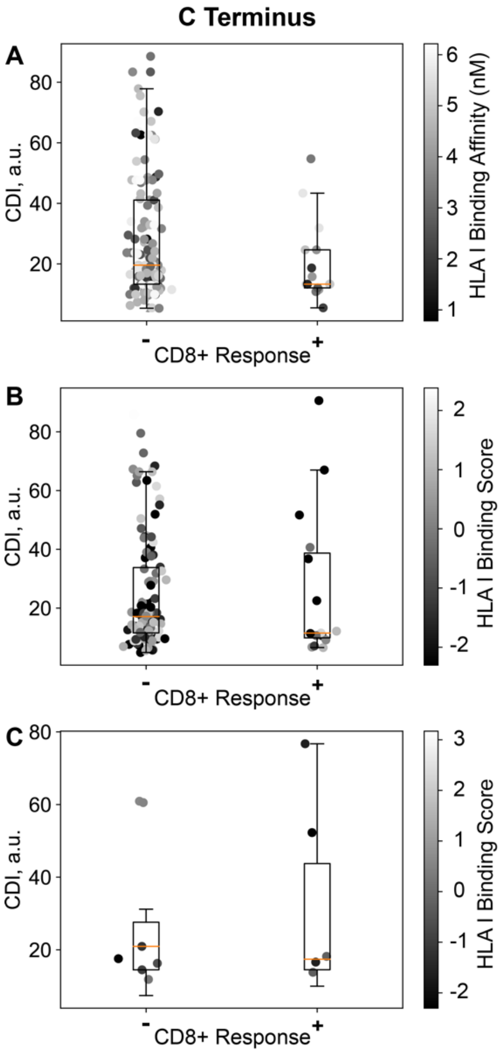

Fig. S29. Retrospective analysis of clinically studied vaccine sequences, which were evaluated for potential proteasomal degradation activity based on C-degron stability: (A) personalized peptide vaccines for melanoma; (B) personalized RNA vaccines for melanoma; and (C) personalized peptide vaccines for glioblastoma. CDI results are graphed using box plots (left Y axis); Individual data points are shaded based on reported binding affinity to HLA molecules (right Y axis). The prediction results were divided into two groups based on the successful (+) or unsuccessful (-) detection of a CD8+ T cell response after vaccination.

II. Materials and Methods

Materials

Fmoc-protected L-amino acids used for peptide synthesis were purchased from Novabiochem. Peptide synthesis couplings were performed with 1-[bis-(dimethylamino)methylene]-1*H*-1,2,3-triazolo[4,5-b]-pyridinium 3-oxid hexafluorophosphate (HATU) and (7-azabenzotriazol-1-yloxy)tripyrrolidinophosphonium hexafluorophosphate (PyAOP), which were purchased from P3 Biosystems. *N*,*N*-Dimethylformamide, piperidine, diisopropylethylamine, trifluoroacetic acid, and triisopropylsilane were purchased from VWR or Millipore Sigma. Antibodies for flow cytometry were purchased from BioLegend. Media for cell culture were purchased from ThermoFisher Scientific. Tissue culture was performed with RPMI 1640 Medium, GlutaMAX™ Supplement, Fetal Bovine Serum, qualified, One Shot™ format. Penicillin-Streptomycin (10,000 U/mL) was purchased from ThermoFisher Scientific. Western blots were performed with nitrocellulose membranes (GE), filters (Bio-Rad Laboratories, Inc.), and PBS blocking buffer (LI-COR Biosciences). Primary and secondary antibodies for visualization of the bands include: Erk1/2 (Cell Signaling), goat anti-mouse IRdye680 (LI-COR Biosciences), and streptavidin IRdye680 (LI-COR Biosciences).

General Equation

The following equation uses: a linear combination of bin populations (e.g., bin1, bin2, bin3, and bin4), which was obtained from Elledge and coworkers; exponentially increasing coefficients (e.g., 0, 1, 10, and 100), which reflect the exponential scale of the original data (i.e., flow cytometry). The resulting equation, which we call the C-terminal Degron Index (CDI), relates proteasomal degradation activity to a numerical score that ranges from 0 to 100.

$$\mathrm{CDI} = bin1 \times 0 \; + \; bin2 \times 1 \; + \; bin3 \times 10 \; + \; bin4 \times 100$$

Synthesis and purification of antigen peptides

Peptides were synthesized on a 0.1 mmol scale by automated flow peptide synthesis. Peptide synthesis was performed on ChemMatrix resin with a 4-(4-Hydroxymethyl-3-methoxyphenoxy)butyric acid (HMPB) linker (200 mg, 0.5 mmol/g, 100–200 mesh). The first amino acid (1 mmol, 10 equiv.) was manually coupled to the resin with DIC (0.5 mmol, 78 µL) and DMAP (0.01 mmol, 50 µL of a 0.2M solution in DMF) in 3.17 mL of DMF. The resin suspension was incubated overnight (16–24 h), then was drained and rinsed three times with DMF (5 mL). Subsequent amino acids were added by automated flow peptide synthesis. After the syntheses were complete, peptide cleavage and global deprotection was performed with a solution of trifluoroacetic acid, water, ethane dithiol, and triisopropyl silane (94/2.5/2.5/1). Purification was achieved by preparative RP-HPLC with Agilent Zorbax SB-C18 Prep HT column (21.2 mm × 250 mm, 7 µm) at a flow rate of 15 mL/min using a gradient with water and acetonitrile containing 0.1% TFA. Pure HPLC fractions were pooled and lyophilized. The purified peptides were analyzed as 0.01 mg/mL solutions (50:50 CH_3CN in H_20 with 1% formic acid) by LC/MS on an Agilent 6550 ESI-Q-TOF mass spectrometer equipped with an Agilent Zorbax 300SB-C3 column (2.1 mm × 150 mm, 5 µM) with a 1–91% gradient of CH_3CN in H_2O with 0.1% formic acid and a flow rate of 0.5 mL/min.

Protein expression and purification

Protective antigen (PA). This protein was expressed in *B. anthracis* strain BH500 from a pYS5 plasmid, which gave PA in high yields with limited endotoxin. Cultures containing plasmid were grown in FA medium containing 10 µg/mL of kanamycin at 37 ˚C for 14 h. The cultures were cooled and supplemented with 2 µg/mL of AEBSF [4-(2-aminoethyl)-benzenesulfonylfluoride HCl], and then centrifuged at 4550 g for 30 min. All subsequent steps

were performed at 4 ˚C. The supernatants were filter sterilized and supplemented with 5 mM EDTA. Solid ammonium sulfate was added to the supernatants to obtain 40% saturation. Phenyl-Sepharose Fast Flow (low sub) (GE Healthcare Life Sciences, Uppsala, Sweden) was added and supernatants gently mixed at 4 ˚C for 1.5 h. The resin was collected on a fritted-disk funnel and washed with buffer containing 1.5 M ammonium sulfate, 10 mM Tris HCl, and 1 mM EDTA (pH 8.0). Protein was eluted with 0.3 M ammonium sulfate, 10 mM Tris HCl, and 1 mM EDTA (pH 8.0), precipitated by adding an additional 30 g ammonium sulfate per 100 mL eluate, and centrifuged at 18,370 g for 20 min. Protein was resuspended in 5 mM HEPES, 0.5 mM EDTA (pH 7.5), followed by loading onto a Q-Sepharose Fast Flow column (GE Healthcare Life Sciences). Protein was eluted with a 0–0.5 M NaCl gradient in 20 mM Tris–HCl, 0.5 mM EDTA (pH 8.0). Protein-containing fractions were identified by SDS-PAGE at 165 V for 36 min on an Invitrogen Bolt™ 4–12% Bis-Tris Plus Gel with Bolt™ MES SDS Running Buffer (1x). Gels were visualized by SimplyBlue™ SafeStain (Coomassie). The clean fractions were pooled and buffer-exchanged into 10 mM Tris, pH 7.5, 150 mM NaCl, and 0.5 mM EDTA. Protein was concentrated as necessary, flash-frozen in liquid nitrogen, and stored at –80 ºC. The exact mass of the purified protein was confirmed by LC/MS on an Agilent 6550 ESI-Q-TOF mass spectrometer equipped with an Agilent Zorbax 300SB-C3 column (2.1 mm × 150 mm, 5 μM) with a 1–91% gradient of CH_3CN in H_2O with 0.1% formic acid and a flow rate of 0.5 mL/min.

N-terminus of Lethal Factor (LF_N). This protein was expressed in BL21(DE3) *E. coli*, which was purchased from New England Biolabs. The protein was expressed at New England Regional Center of Excellence/Biodefense and Emerging Infectious Diseases (NERCE) and was purified. LF_N was expressed as the SUMO-LF_N-LPSTGG-H_6 construct in a Champion pET-SUMO vector. LF_N was isolated from *E. coli* pellets by suspension in Tris buffer (20 mM Tris, 150 mM NaCl,

pH 8.5), lysis by sonication, and purification with a HisTrap FF Ni-NTA column. Purified fractions of PA and LF_N were analyzed by SDS-PAGE at 165 V for 36 min on an Invitrogen Bolt™ 4–12% Bis-Tris Plus Gel with Bolt™ MES SDS Running Buffer (1x). Gels were visualized by SimplyBlue™ SafeStain (Coomassie). Clean fractions were pooled and concentrated with Amicon® Ultra-15 Centrifugal Filter Units.

Sortase-mediated ligations

Semi-synthetic LF_N protein constructs were prepared by enzymatic ligation using SrtA*. LF_N-LPSTGG-H_6 (80 μM) was combined with synthetic peptides (800 μM) in PBS buffer (pH 8.5, endotoxin free, Corning), in addition to adding a 50x dilution of 0.5 M $CaCl_2$ reaction buffer (freshly prepared, endotoxin free) to give a final concentration of 10 mM $CaCl_2$. The reaction mixture was gently rotated for 45 min, followed by adding triple-rinsed Ni-NTA agarose beads (50 μL per mg of protein), which enabled isolation of the enzyme: H_6-SrtA*; reacted starting material: GG-H_6; and unreacted starting material: LF_N-LPSTGG-H_6. Collection of the reaction supernatant was achieved by centrifugation of the reaction mixture (30 s × 16,000 rpm), followed by successive rounds of rinsing with PBS (3 × 0.5 mL). To the combined rinses was added aqueous EDTA (0.5 M, pH 7.5, 100 μL, freshly prepared, endotoxin free) to sequester the $CaCl_2$. The mixture was pushed through a 0.2 μm syringe filter, then buffer exchanged using Amicon® Ultra-15 Centrifugal Filter Units (MSCO = 30 kDa) to remove the excess peptide. The purified peptide-conjugated LF_N was analyzed by SDS-PAGE at 165 V for 36 min on an Invitrogen Bolt™ 4–12% Bis-Tris Plus Gel with Bolt™ MES SDS Running Buffer (1x). Gels were visualized by SimplyBlue™ SafeStain (Coomassie). The exact mass of the purified protein was confirmed by LC/MS on an Agilent 6550 ESI-Q-TOF mass spectrometer equipped with an Agilent Zorbax

300SB-C3 column (2.1 mm × 150 mm, 5 μM) with a 1–91% gradient of CH_3CN in H_2O with 0.1% formic acid and a flow rate of 0.5 mL/min.

LC-MS protein characterization

Protein (50 ng) was loaded onto an Agilent Zorbax 5 μm 300SB-C3 column (2.1 × 150 mm) and was eluted with a gradient of 1–91% CH3CN in H2O with 0.1% FA and a flow rate of 0.5 mL/min. The protein was detected on an Agilent 6550 ESI-Q-TOF mass spectrometer.

Western blot

Proteasome-mediated degradation was examined by Western Blot analysis. These experiments were performed by plating CHO-K1 cells at 2×10^5 cells/well in a 12-well tissue culture plate. After incubating for 16 h at 37 ºC and 5% CO_2, the cells were resuspended in media treated with or without 50 μM lactacystin. After incubating for 1 h, the cells were resuspended in media containing PA (50 nM) and an LF_N construct (LF_N **1**–**3**). After incubating for 24 h, the wells were washed with PBS and trypsonized (0.25% trypsin–EDTA) for 5 min at 37 ºC and 5% CO_2. The cell pellets were treated with lysis buffer (50 μg/mL digitonin, 75 mM NaCl, 1 mM NaH_2PO_4, 8 mM Na_2HPO_4, 250 mM sucrose, and Roche cOmplete^{TM} protease inhibitor cocktail, pH 7.7) for 10 min on ice, then pelleted for 5 min at 4 ºC and 16,000 rcf. The lysates were filtered using AcroPrep Filter Plates with Bio-Inert Membranes (Pall Life Sciences), then separated by SDS-PAGE and transferred onto Western Blot membranes. The membranes were developed using optimized conditions from prior expeirments (not shown). Membrane blocking was achieved by incubating for 1 h in PBS containing 5% LI-COR blocking buffer. Primary antibodies were incubated for 2 h in a solution of TBST (0.05%) and LI-COR blocking buffer (5%) containing the following antibodies: anti-ERK1/2 (Cell Signaling Technologies; dilution factor: 1/2000) and streptavidin IRdye680 (LI-COR Biosciences; dilution factor: 1/2000). The goat anti-rabbit

IRdye680 secondary antibody (LI-COR Biosciences; dilution factor 1/5000) was incubated for 1 h in TBST (0.05%) and LI-COR blocking buffer (5%). The membrane was imaged on an Odyssey Imaging System (LI-COR Biosciences).

Endotoxin testing and removal

Protein endotoxin levels were measured at 0.045 μg/mL using single-use cartridges (0.05 EU/mL, PTS2005) and the Endosafe® nexgen-PTS™ reader (Charles River). If endotoxin levels were > 0.015 EU/μg, established procedures to remove endotoxin were followed. In particular, Pierce™ High-Capacity Endotoxin Removal Resin was used to reduce endotoxin levels for peptide-conjugated LF_N constructs. Prior to use, a 1-mL aliquot of resin was centrifuged (14000 rpm × 5 min) and resuspended in an equivalent volume of endotoxin-free phosphate-buffered saline (PBS). The resuspended resin was then added to each protein sample at a 1:4 or 1:8 v/v ratio (resin volume/protein volume). After mixing gently for 1 h at room temperature, the resin was removed by filtering the samples through a 0.2 μm filter. This procedure provided high protein recovery (~90%) and was repeated (1–3x) until sufficient endotoxin levels were reached (≤ 0.015 EU/μg).

Murine immune response

Animal studies were carried out under an institute-approved IACUC protocol following federal, state, and local guidelines for the care and use of animals. C57BL6/J and OT-1 mice were procured from The Jackson Laboratory. Six- to 12-week-old female mice were used for these studies. Antibodies for flow cytometry studies were purchased from Biolegend: CD69 (clone H1.2F3); CD137 (clone 17B5); CD3 (clone 17A2); CD90.1 (clone OX-7); and CD8a (clone 53-6.7). Experiments with murine splenocytes utilized freshly harvested spleens from naïve mice. Dendritic cells were isolated with the EasySep™ Mouse Pan-DC Enrichment Kit II (Stem Cell

Technologies). T Cells were isolated with the EasySep™ Mouse T Cell Isolation Kit (Stem Cell Technologies). Antibody staining was performed at a dilution of 1:100 for 25 minutes at 4 ºC in the presence of mouse Fc block (TruStain FcX™, anti-mouse CD16/32, BioLegend) in PBS containing 5% FBS. Cell proliferation was monitored using CellTrace™ Violet Cell Proliferation Kit (ThermoFisher Scientific). Viability was assessed by LIVE/DEAD Fixable Aqua (Life Technologies). Cells were analyzed using BD LSR Fortessa. Data were analyzed using FlowJo v10. Peptides for restimulation were used at a concentration of 10 mg/mL with sequences as follows: $OVA_{257–264}$, SIINFEKL and $gp100_{20–33}$, EGPRNQDWL.

Mixed lymphocyte reaction

Murine splenocytes were freshly isolated from mouse spleens by homogenization over a mesh filter (100 μm) in RPMI 1640 Medium (GlutaMAX™ Supplement, 10% Fetal Bovine Serum, and 1% Penicillin-Streptomycin). Single-cell suspensions were created by re-filtering the cells through a cell strainer (45 μm). For C57Bl/6 mice, dendritic cells (DCs) were enriched by magnetic isolation using the EasySep™ Mouse Pan-DC Enrichment Kit (Stem Cell Technologies). DCs were plated into 96-well U-bottom plates (100×10^6 cells/well), followed by incubation with or without lactacystin (20–100 μM), at 37 ºC and 5% CO_2. After 4 h, the DCs were centrifuged (5 min. × 400 rcf) and resuspended in the protein treatments. The DCs were incubated with the proteins for 1 h, followed by centrifugation and resuspension in T cells (50×10^6 cells/well). During the DC incubations, T cells from the transgenic mouse splenocytes were enriched by magnetic isolation using the EasySep™ Mouse T Cell Isolation Kit (Stem Cell Technologies). T cells were centrifuged and resuspended in PBS, followed by treatment with CellTrace™ Violet Cell Proliferation Kit (ThermoFisher Scientific) according to the manufacturer's protocol. T cells were centrifuged and resuspended in RPMI media, plating with the DCs. After 24 h, upregulation

of early-activation markers (CD69 and CD137) were evaluated. After 72 h, the magnitude of the T cell proliferation was evaluated by flow cytometry.

Statistical analysis

Results from mixed lymphocyte reactions were analyzed using GraphPad Prism software. Data from protein constructs were grouped within the same experiment, then compared with the indicated statistical analyses.

Representation of peptides

The peptides were represented as a matrix of extended connectivity fingerprints of individual amino acids, generated using RDKit (http://www.rdkit.org/). For the individual fingerprints, we used a radius of 3, and 128 bit-size. For the peptides, we considered only the last 24 amino acids, and used 'left' padding for shorter sequences.

Machine learning

We developed a multi-layer Conv1D model using TensorFlow and optimized the hyperparameters over 1000 iterations using SigOpt (https://sigopt.com/). The hyperparameter optimization minimized the RMSE as the objective function. After the hyperparameter optimization, we used the hyperparameters for the top 5 models, and re-trained 5 models each with different random seeds. Finally, we used 25 models (5 top-hyperparameter x 5 random-seed) for the prediction, analysis and screening of different peptides.

Genetic algorithm for generation of new peptides

We used directed evolution to generate new peptides. For peptides of different lengths, we used a random seed of the desired length from the training dataset as the C-terminus degron, keeping the N-terminus and epitope constant. Then, we performed randomized single and multi-site swapping of amino acids. In the single-site swapping, we randomly selected a particular

position at the C-terminus and replaced it with a random residue. Similarly, in the multi-site swapping, we selected a string of amino acids and replaced it with another random string from the training dataset with the same length. The objective of the genetic algorithm was to increase the CDI score.

Selection of peptides for experimental evaluation

As a part of the genetic algorithm-based optimization, we obtained new peptides having a wide range of predicted CDI scores. We also calculated the Grand Average of Hydropathy (GRAVY) score and iso-electronic points (pI) to infer ease-of-experimental synthesis and purification. The new peptides were filtered with a GRAVY score of <= 0.1, and with a pI either between 4 and 6.5 or between 7.5 and 10.
